# Thermally Evaporated Naphthalene Diimides as Electron Transport Layers for Perovskite Solar Cells

**DOI:** 10.1021/acs.chemmater.5c01186

**Published:** 2025-08-26

**Authors:** Jack Lawton, Justine S. Wagner, Xiangyu Xiao, Sanggyun Kim, Anna M. Österholm, D. Eric Shen, Sina Sabury, Carlo A. R. Perini, Kunal Datta, Diana K. LaFollette, Ruipeng Li, John R. Reynolds, Juan-Pablo Correa-Baena

**Affiliations:** † School of Materials Science and Engineering, 1372 Georgia Institute of Technology, North Ave NW, Atlanta, Georgia 30332, United States; ‡ Georgia Institute of Technology School of Chemistry and Biochemistry, Georgia Institute of Technology, Atlanta, Georgia 30332, United States; § National Synchrotron Light Source II, 8099 Brookhaven National Laboratory, Upton, New York 11973, United States of America

## Abstract

Thermally evaporated organic electron transport layers (ETLs) have the potential to enable high-performance and scalable perovskite solar cells (PSCs). Among these, naphthalene diimide (NDI)-based ETLs are a promising family of materials that exhibit the optoelectronic properties, ambient stability and versatility required of high-performance ETLs. Here, we synthesized five NDI derivatives with varying functional groups and identified the two most promising candidates for evaluating the impact of molecular structure on processability via thermal evaporation. While phosphonic acid functionalization was shown to introduce thermal instability, leading to chemical changes during evaporation, NDI-bis *N*-phenyl-bromide (NDI-(PhBr)_2_) emerged as a promising ETL candidate. NDI-(PhBr)_2_ demonstrated excellent compatibility with the thermal evaporation process and enabled PSCs with power conversion efficiencies (PCEs) of 15.6%, surpassing all previously reported PSCs containing thermally evaporated NDI ETLs. Furthermore, NDI-(PhBr)_2_ exhibited excellent operational stability, retaining 75% of the initial PCE after 150 h of operation under continuous illumination at 65 °C. These results highlight the potential of NDI-based ETLs for advancing the scalability and performance of PSCs.

## Graphical abstract

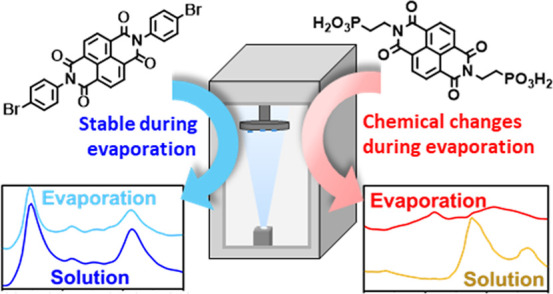

## 1 Introduction

Perovskite solar cells (PSCs) have exhibited a rapid increase in power conversion efficiency (PCE) since their inception over a decade ago.^1^ The excitement around PSCs has been bolstered by their rapid advancement and the promise of low-cost and scalable device fabrication. The electron transport layer (ETL) is an essential component of a high-performing PSC that facilitates the extraction and transport of electrons from the perovskite layer to the electrode, while simultaneously blocking holes.^2^ Traditionally, inorganic ETLs such as TiO_2_ and SnO_2_ have been used, but recent focus has shifted toward organic ETLs due to their low processing temperatures, chemical adaptability and compatibility with thermal evaporation.^3,4^ However, there are few reports on the development of new organic ETLs that can be processed via both solution and evaporation. The most commonly used organic ETLs are fullerene-based materials, such as C_60_ and [6,6]-phenyl-C_61_-butyric acid methyl ester (PCBM).^5,6^ Despite advantageous charge transport properties, these materials suffer from poor frontier orbital tunability, high synthetic costs, and poor ambient stability, which has motivated the development of nonfullerene-based organic ETLs.^7^ Naphthalene diimide (NDI) and its derivatives provide an alternative route to achieving high efficiency PSCs that incorporate organic ETLs. NDIs have been extensively used in organic electronics owing to their electron affinity, high electron mobilities, tunable optical absorption profiles, and exceptional thermal stability.^8^ The strong electron withdrawing nature of imide functionalities results in NDIs possessing an electron deficient conjugated core, allowing it to form relatively stable radical anions.^9^ Cyclic voltammetry or a combination of ultraviolet photoelectron spectroscopy and UV–vis spectroscopy have been used to show that NDI materials exhibit a deep lying LUMO, often within close proximity to the perovskite conduction band.^10–14^ This allows NDI-based materials to act as n-type organic semiconductors, making them a prime candidate for use as organic ETLs.^15^ The structural versatility of NDI molecules has spurred extensive research, leading to an array of derivative molecules functionalized at both the axial and terminal positions, as well as the integration of NDI subunits into polymers.^16–20^

A key challenge limiting the widespread integration of NDI-based materials into conventional PSC architectures is the poor solubility of the parent NDI, which has terminal hydrogens on the imide nitrogen (NDI-H).^21^ One strategy to address the insolubility in common processing solvents leverages the structural tunability of NDIs by introducing solubilizing side groups. These structural modifications are effective in enhancing solubility, but some functionalization attempts, such as axial *tert*-butoxycarbonyl groups have been found to disrupt the optimal face-on stacking of the NDI cores that is desired for efficient charge transport.^14^ Despite this, functionalization using fluorinated hydrocarbons or chiral groups has enabled devices containing NDI-based ETLs to be competitive with the state-of-the-art perovskite technologies.^13,22^

While undoubtedly impressive results have been obtained for PSCs utilizing NDI-based ETLs deposited via spin coating, incompatibility with large area or textured substrates has driven research into alternative solvent-free deposition techniques, such as thermal evaporation. Thermal evaporation involves the sublimation of precursor materials under high vacuum and circumvents the need for processing solvents. This technique enables uniform coating of thin films, with precise control over film thickness and stoichiometry, while being well-suited for industrial scale-up.^23^ However, not all organic materials are compatible with the thermal evaporation process, as sufficient thermal stability is required at the elevated temperatures necessary for sublimation. It has been demonstrated that certain functional groups can, on occasion, render an otherwise evaporation-stable material unstable.^24,25^ A notable example of this is that while C_60_ can be readily sublimed, PCBM undergoes multiple decomposition routes when heated in ultrahigh vacuum, resulting in thermally evaporated films containing PCBM, C_60_, and other decomposition products.^26^ Limited research has been conducted on the thermal evaporation of NDIs for PSCs. Previously, unfunctionalized NDI-H was thermally evaporated as an ETL, achieving power conversion efficiencies of 10% and outperforming the solution-processed analog.^14^ Currently, there are few reports on how NDI functionalization influences the stability, structure, and performance of thermally evaporated NDI thin films. Gaining a deeper understanding of this relationship is essential for bridging the performance gap between spin coated and evaporated NDI films and could serve as a crucial steppingstone in achieving high-performance, scalable PSCs.

In this work, we introduce the synthesis of five NDI derivatives designed to explore the impact of functional groups on the processability of NDI molecules via thermal evaporation. Of the five synthesized molecules, we identifed two NDI molecules to study in depth: one molecule functionalized with the commonly used phosphonic acid group ethyl phosphonic acid (NDI-(EtPA)_2_) and another with 4-bromophenyl (NDI-(PhBr)_2_) terminations. The former is expected to form strong interactions with the oxide substrate, whereas the latter is not. The molecule selection rationale is directed to understanding how terminal groups in these small molecules affect their deposition via thermal evaporation. The NDI-(PhBr)_2_ was found to be stable during thermal evaporation, while NDI-(EtPA)_2_ underwent chemical changes during evaporation. The chemical composition and structure of both films were gauged via X-ray photoelectron spectroscopy (XPS), Fourier transform infrared spectroscopy (FTIR), and grazing incidence wide-angle X-ray scattering (GIWAXS). NDI-(PhBr)_2_ was shown to maintain its molecular structure during evaporation, while structural changes were observed in NDI-(EtPA)_2_. Despite the observed thermal instability, structural analysis confirmed that NDI cores were still present in the evaporated NDI-(EtPA)_2_ films. Optical absorption measurements demonstrated that both films possessed a suitable large bandgap for use as an ETL in an n–i–p device. The sensitivity of the NDI films to processing solvents used during perovskite deposition was also investigated. We found that, for both molecules, a sufficient amount of material remained after exposure to polar solvents, which enabled them to act as effective ETLs. Finally, devices were fabricated to test the performance and long-term stability of NDI-(PhBr)_2_ and NDI-(EtPA)_2_. The devices exhibited PCEs of 15.6% and 14.1% for NDI-(PhBr)_2_ and NDI-(EtPA)_2_, respectively. Additionally, our devices were shown to be stable to environmental stressors such as heat, oxygen, and ultraviolet light and maintained a stable PCE over an operational period of 150 h.

## 2 Results and Discussion

### 2.1 Molecular Synthesis and Thermal Stability

To enable ETL deposition via thermal evaporation, it was essential to identify a set of molecules that not only met the key requirements for an effective ETL in a PSC but also possessed the thermal stability necessary for successful evaporation. We synthesized five NDI derivatives functionalized only at the terminal imide positions, as shown in Figure 1A. The success of each synthetic step and purity of the final products were confirmed via ^1^H, ^13^C, and ^31^P nuclear magnetic resonance (NMR) spectroscopy (see Figures S1–S31). Functionalization with ethyl-phosphonic acid was chosen for its ability to form robust covalent bonds with oxide substrates, promoting strong adhesion and minimizing delamination.^27^ Additionally, previous research has shown that phosphonic acids can induce the growth of cubic formamidinium (FA) based perovskites phases, leading to improvements in device performance.^28,29^ Aliphatic hexyl chains were selected to improve solubility by virtue of their conformational flexibility, which allows for easier synthesis, purification, and isolation. Aryl-bromide groups were selected to enhance the solubility relative to NDI-H, however, they offer greater rigidity than alkyl chains, which could improve thermal stability. The hydrophobic character of the bromophenyl and hexyl chains, relative to ethyl phosphonic acid, could potentially also enhance long-term stability under humid conditions.^30^

**Figure 1:**
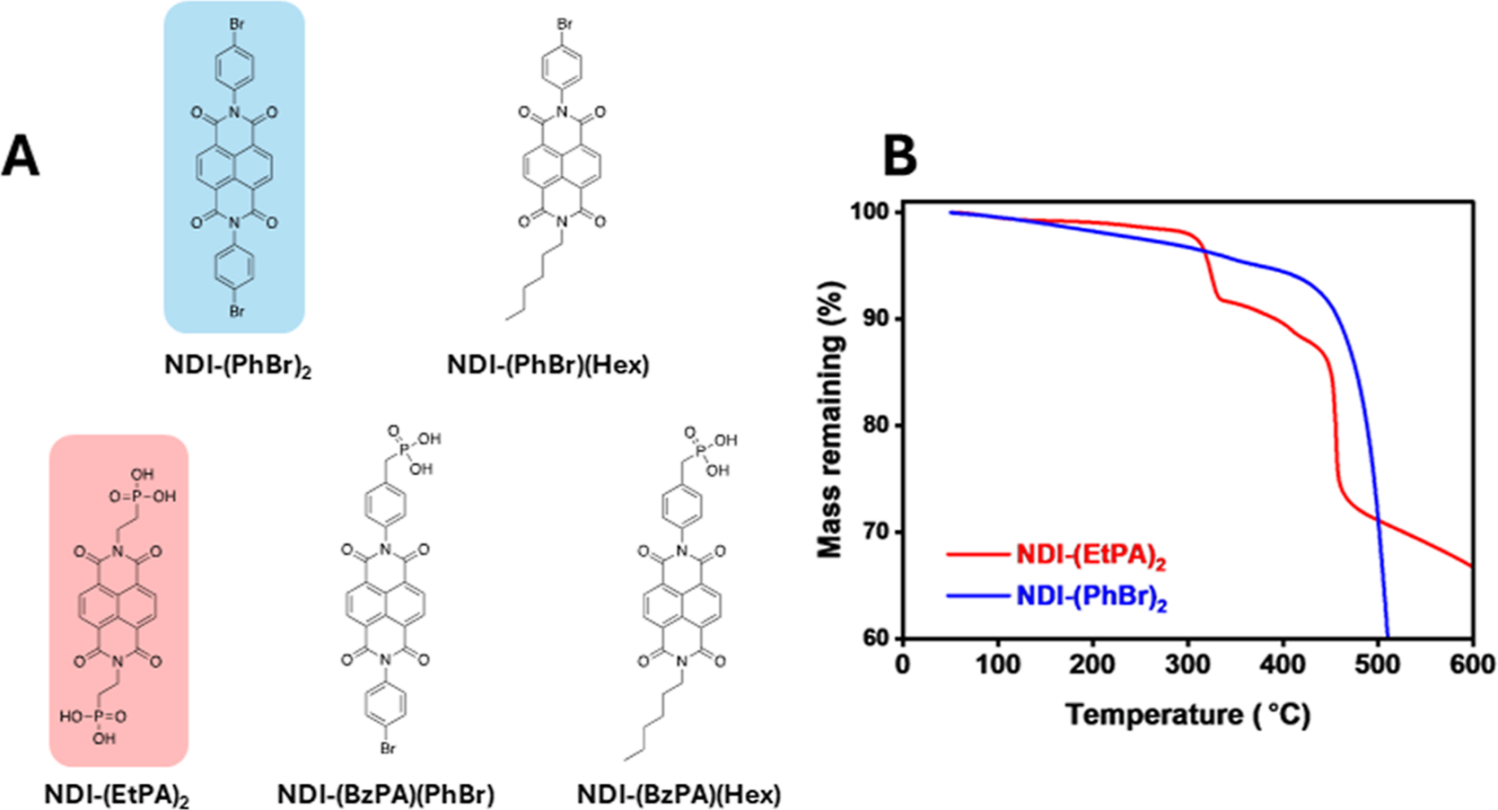
(A) Chemical structures of the five synthesized molecules, including the highlighted molecules NDI-(EtPA)_2_ (red) and NDI-(PhBr)_2_ (blue) which are the focus of this paper. (B) Thermogravimetric analysis of NDI-(EtPA)_2_ (red) and NDI-(PhBr)_2_ (blue), measured at a ramp rate of 15 °C/min under a N_2_ atmosphere.

Given the focus on the thermal evaporation of the NDI molecules, understanding their thermal stability is essential. For a molecule to undergo complete evaporation without decomposition, sufficient thermal energy must be provided to overcome the intermolecular forces present while avoiding bond cleavage and thermal degradation.^31^ Thermogravimetric analysis (TGA) provides valuable insights into a material’s suitability for evaporation by highlighting the thermal instabilities of a material. Figure 1B shows the thermograms for both NDI-(EtPA)_2_ and NDI-(PhBr)_2_ under N_2_ atmosphere, while thermograms for the other three NDI derivatives are provided in Figure S32. For NDI-(PhBr)_2_ (Figure 1B) and NDI-(PhBr)­(Hex) (Figure S32), which lack phosphonic acid functionalization, a single distinct mass loss event occurs with an onset temperature of approximately 440 °C. In contrast, the thermogram of NDI-(EtPA)_2_ (Figure 1B) exhibits a more complex profile, showing an initial mass loss of approximately 10% at 315 °C followed by a second mass loss event at around 440 °C. Given that neither of these mass loss events result in complete volatilization, they likely correspond to some decomposition process, either via the expulsion of volatile products or the formation of side products within the material. A purely evaporative process would be characterized by a large single-step mass loss. Similar multiple-step mass loss behavior is observed for NDI-(BzPA)­(Hex) and NDI-(BzPA)­(PhBr) (Figure S32), indicating that this instability is an inherent characteristic of NDI molecules functionalized with phosphonic acid groups. Furthermore, the similarity in the thermograms of NDI-(EtPA) and NDI-(BzPA)­(PhBr) suggests that replacing the ethyl linker with a benzyl linker does not significantly improve the thermal stability of the molecule. The observed thermal instability of PA-functionalized NDI molecules underscores the need for careful consideration of functional group selection when designing NDI derivatives for thermal evaporation processes.

### 2.2 Characterization of Thermally Evaporated Thin Films

To further probe the thermal evaporation and subsequent performance of NDI-based ETLs, we selected NDI-(PhBr)_2_ and NDI-(EtPA)_2_ for further investigation as their distinct, yet different, thermal behaviors can provide valuable insight into the impact of different terminal substituents on the evaporation characteristics. The single, high-temperature mass loss event observed for NDI-(PhBr)_2_, indicating clean evaporation with minimal decomposition, makes it a strong candidate for processing via thermal evaporation. In contrast, NDI-(EtPA)_2_, while exhibiting multiple mass loss events, serves as a representative of the PA-functionalized NDIs, allowing for direct comparisons of how these functional groups influence thermal stability. By focusing on these two symmetrically substituted molecules, we minimize the complexities introduced by asymmetry, facilitating a cleaner understanding of structure–property relationships critical for optimizing thermally evaporated ETLs.

Thin films of the NDI molecules were deposited via thermal evaporation on fluorine-doped tin oxide (FTO) coated glass, glass slides, and flexible poly­(ethylene terephthalate) substrates coated with indium-doped tin oxide (PET:ITO). A schematic of the evaporation chamber used can be seen in Figure S33. Each evaporation was monitored via a quartz crystal microbalance (QCM) and experimental parameters such as pressure, deposition rate, and temperature are shown in Figures S34 and S35. The evaporation of both molecules continued until QCM 1 stopped registering the deposition of fresh material. Both NDI-(PhBr)_2_ and NDI-(EtPA)_2_ exhibited distinct deposition events, as shown in Figures S34 and S35, at approximately 220 and 380 °C, respectively. The elevated deposition onset temperature of NDI-(EtPA)_2_ could be explained by the stronger intermolecular forces that arise from the phosphonic acid-induced hydrogen bonding. The deposition temperature of NDI-(PhBr)_2_ was recorded to be below that of the first mass-loss event, which was shown to be approximately 440 °C. This is a strong indication that NDI-(PhBr)_2_ possesses sufficient thermal stability to be thermally evaporated. Conversely, the thermogram of NDI-(EtPA)_2_ in Figure 1B exhibits a mass loss event at 315 °C. This is below the temperature at which deposition was recorded (Figure S34), which suggests that NDI-(EtPA)_2_ does not possess the thermal stability to evaporate without undergoing some form of chemical change. After thermal deposition, no material remained in the crucible after the evaporation of NDI-(PhBr)_2_, while residual material was found in the crucible containing NDI-(EtPA)_2_ (Figure S36). In fact, for all molecules containing phosphonic acid functionalities, some degree of material remained in the crucible post deposition. This corroborates our hypothesis that phosphonic acid groups have a propensity to undergo some degree of chemical change during evaporation, when functionalized to an NDI core. While reports on thermally evaporated small molecule organic ETLs are sparse, thermally evaporated hole transport layers (HTLs) are better studied. One example is the [2-(9*H*-carbazole-9-yl)­ethyl]­phosphonic acid (2PACz) family of molecules, which consists of phosphonic acid-functionalized carbazoles.^32^ Thermal instability has been identified in this family of molecules, whereby degradation and residual powders were identified at temperatures above 200 °C.^33^ Our findings, alongside these reports, lead us to conclude that ethyl phosphonic acid functionalization introduces inherent thermal instability to certain small molecules. Therefore, emphasis should be placed on the future choice of the phosphonic acid-functionalized core molecule and the temperatures at which they are thermally evaporated to ensure that film deposition can occur before chemical changes happen to the parent molecule.

To identify changes in the chemical structure of these NDI molecules, we can compare thermally evaporated films to drop-cast films, given that no molecular changes are to be expected during room-temperature solution processing of the NDI molecules. Attenuated total reflectance Fourier transform infrared (FTIR) spectroscopy can help identify any chemical changes within a material by identifying changes in molecular vibrations, which relate to changing chemical environments and the cleavage/formation of chemical bonds. Figure 2A shows the FTIR spectra for thermally evaporated and solution processed films of NDI-(EtPA)_2_ and NDI-(PhBr)_2_ on flexible PET:ITO substrates. The spectra for both evaporated and solution processed NDI-(PhBr)_2_ share identical peak distributions, with some variation in peak intensity. This suggests that NDI-(PhBr)_2_ is evaporated without thermal decomposition taking place. When considering the FTIR spectra of the evaporated and solution processed NDI-(EtPA)_2_ thin films, distinct differences are evident. Figure S37 provides an overlay of the FTIR spectra of both the solution processed and thermally evaporated thin films of NDI-(EtPA)_2_ and NDI-(PhBr)_2_ between the range of 1550 cm^–1^ and 1300 cm^–1^. Throughout this range several differences in peak intensities can be seen between the NDI-(EtPA)_2_ spectra, signifying structural changes occurring during thermal evaporation.

**Figure 2:**
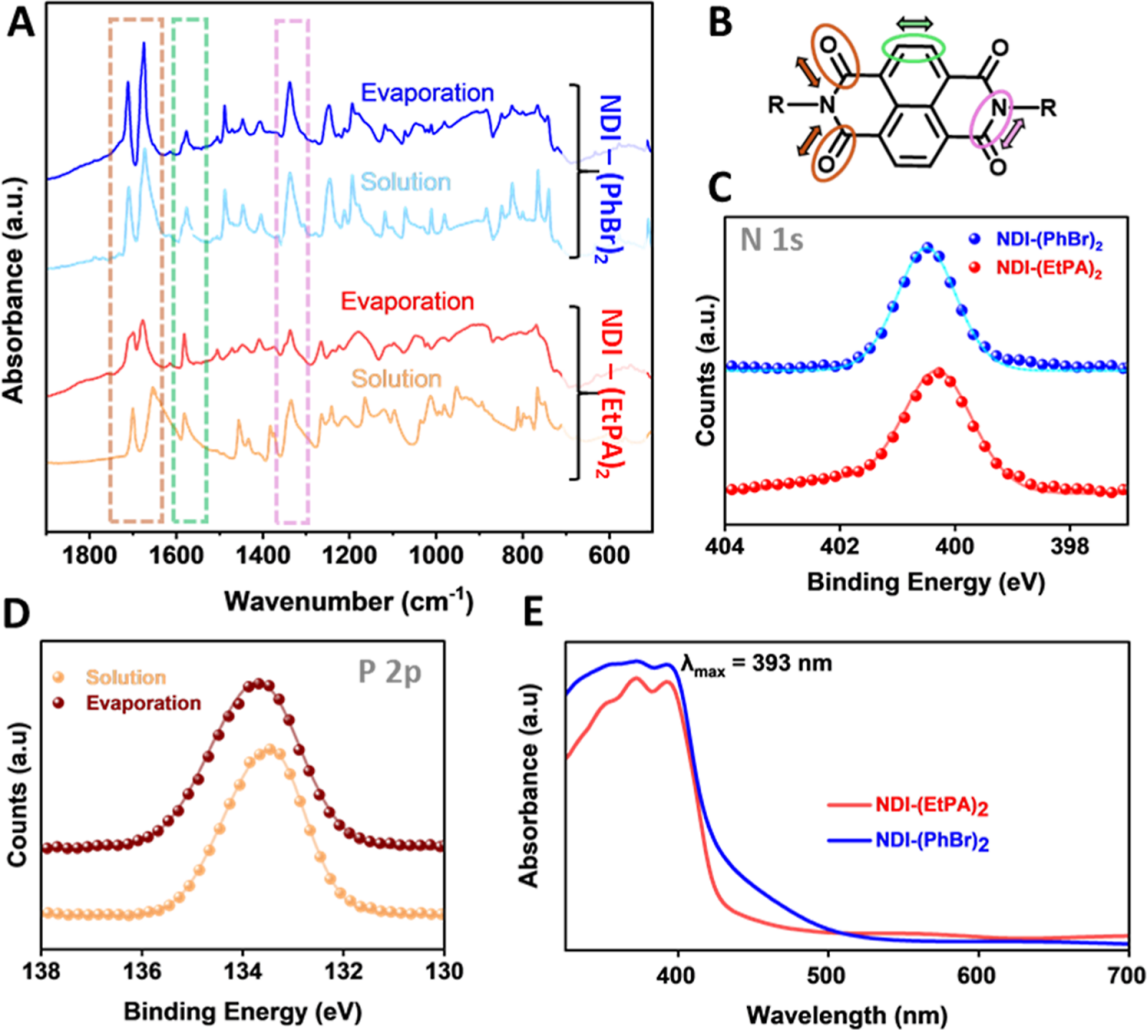
(A) FTIR spectra in the fingerprint region (1900 cm^–1^ to 500 cm^–1^) for thin films of NDI-(PhBr)_2_ (top) and NDI-(EtPA)_2_ (bottom) deposited via evaporation and solution. (B) NDI core with bonds exhibiting identifiable vibrational frequencies highlighted. (C) N 1s XPS spectra for NDI-(PhBr)_2_ (blue) and NDI-(EtPA)_2_ (red). The spheres represent the recorded data, while the lines represent the fitted envelope. (D) P 2p XPS spectra for NDI-(EtPA)_2_ processed via solution (orange) and evaporation (brown). (E) Optical absorption spectra for NDI-(PhBr)_2_ (blue) and NDI-(EtPA)_2_ (red) thin films deposited via thermal evaporation.

Figure 2A compares FTIR spectra of evaporated and solution deposited films which allow us to be more precise in our understanding of the chemical changes occurring during evaporation. First, a doublet is observed at around 1700 cm^–1^ which can be attributed to the symmetric and asymmetric C=O stretches of the imide moiety.^34,35^ While the imide doublets exhibit similar features in the NDI-(PhBr)_2_ spectra, a distinct change is seen in the NDI-(EtPA)_2_ doublet. The thermally evaporated film shows a narrowing of the doublet split, alongside a distinct narrowing of the peak observed at lower wavenumbers which is associated with the asymmetric carbonyl stretch.^36^ This evolution, coupled with the more dramatic changes in Figure S37, suggests chemical changes have occurred, which disrupts the symmetry of NDI-(EtPA)_2_. Additionally, a peak at approximately 1580 cm^–1^ is observed in all spectra and highlighted via the green box in Figure 2A. This can be attributed to the stretching vibration of the naphthalene ring skeleton.^37,38^ A peak is also observed in all spectra at 1335 cm^–1^, as highlighted in the purple box in Figure 2A. This is attributed to the C–N imide stretch.^39,40^ These characteristic vibrations are schematically presented in Figure 2B and suggest that the core NDI-structure is mostly uncompromised during deposition via thermal evaporation for either molecule, along with suggesting that in the case of NDI-(EtPA)_2_ the changes may be occurring on the EtPA moiety. Meanwhile the FTIR analysis supports the argument that NDI-(PhBr)_2_ remains fully intact throughout the evaporation process.

We next conducted X-ray photoelectron spectroscopy (XPS) measurements on NDI films deposited on FTO to provide insights into the chemical bonding and environment present within the NDI films. Figure 2C shows the N 1s spectra for thermally evaporated thin films of both NDI-(PhBr)_2_ and NDI-(EtPA)_2_. The emitted photoelectrons were detected at similar peak positions of 400.5 and 400.3 eV for NDI-(PhBr)_2_ and NDI-(EtPA)_2_, respectively. The proximity of these photoelectron peaks suggests that the majority of the nitrogen exists in the same imide environment for both samples, providing further evidence that the NDI core remains intact during the evaporation of either molecule. The small difference in binding energy can be ascribed to differing electronic environments around the nitrogen in NDI-(PhBr)_2_ and NDI-(EtPA)_2_ due to the presence of an sp^2^ C–N bond and an sp^3^ C–N bond, respectively. Interestingly, a tail at higher binding energies can be observed for NDI-(EtPA)_2_ that is not observed in the solution processed film shown in Figure S38. While it is difficult to ascribe this tail to any particular species, its presence suggests that during the evaporation of NDI-(EtPA)_2_ a minor product is formed in which the nitrogen exists in a more electron deficient environment. Figure 2D shows the P 2p XPS spectra for NDI-(EtPA)_2_ deposited via solution and thermal evaporation. Phosphorus is present on the surface of both samples, and the peak binding energies were found to be 133.5 and 133.6 eV, respectively. The presence of the P 2p XPS signals, along with previously discussed evidence of the NDI core being maintained, makes conclusions about the nature of the thermal instability difficult. While the apparent robustness of the NDI core could lead one to conclude that decomposition is due to the alkyl phosphonate functionality, cleavage of this moiety in its entirety is unlikely, given the relatively small mass loss observed in the early decomposition stage of the NDI-(EtPA)_2_ thermogram that is shown in Figure 1B.

Further insights into the nature of the films deposited by thermal evaporation, and their suitability as charge transport layers, can be found through characterization of their optoelectronic properties. Figure 2E shows the UV–vis absorption spectra for thin films of thermally evaporated NDI-(EtPA)_2_ and NDI-(PhBr)_2_. As expected, both molecules possess the same absorption peak with a maximum value of 393 nm. This matches the absorption values found in the literature for NDI-H and its derivatives attributed to the π–π* transition of the NDI core.^41,42^ This corroborates previous assertions from FTIR and XPS that the NDI core is being evaporated. NDI-(PhBr)_2_ exhibits a lower-energy absorption onset than NDI-(EtPA)_2_, with a gradual absorption increase beginning at approximately 500 nm, whereas NDI-(EtPA)_2_ shows a sharp onset beginning at approximately 430 nm. It is possible that this gradual absorption onset, alongside the reduced vibronic features, is due to a more disordered film in the instance of NDI-(PhBr)_2_ relative to NDI-(EtPA)_2_.^43^ The optical absorption spectra also allow for determination of the optical band gap through the generation of Tauc plots (Figure S39). The bandgaps of NDI-(EtPA)_2_ and NDI-(PhBr)_2_ were found to be 2.96 and 2.95 eV, respectively. These band gaps are sufficiently wide to allow the transmission of visible and NIR light, which is vital for efficient ETLs to maximize the number of photons reaching the active layer and optimize the PCE in solar cells.^44–46^

### 2.3 Thin Film Stability under Operating Conditions

Thermochemical stability is a vital consideration for each layer of the device stack in a perovskite solar cell. Current testing standards require devices to exhibit stability at 85 °C, and hence it is important that all layers within a PSC are thermally robust at these elevated temperatures.^47^ Additionally, films should be able to withstand the synergetic effects of oxygen and UV light to minimize photooxidation. To test the thermal stability of such films in standard operating conditions, FTIR spectra were collected before and after stressing the NDI film at 90 °C for 12 h in air (with around 60% relative humidity) under 1 sun equivalent white light. As can be seen from Figure 3A, there are no significant changes in the vibrational modes of the films before and after stressing. This suggests that these ETL films are structurally stable in ambient operating conditions.

**Figure 3:**
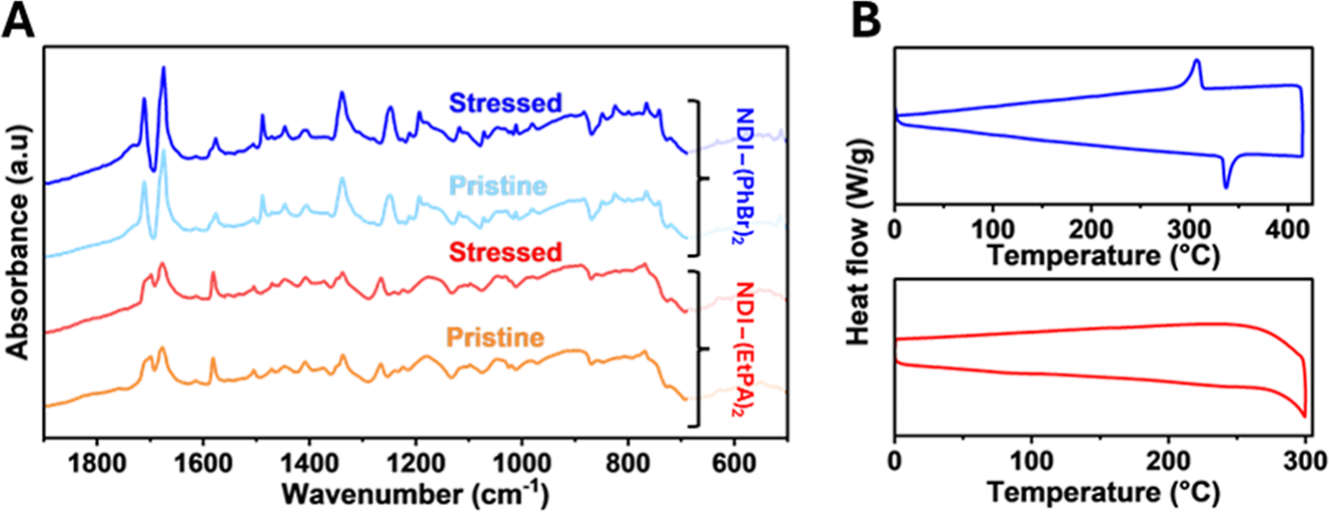
(A) FTIR spectra of NDI-(PhBr)_2_ (blue) and NDI-(EtPA)_2_ (orange/red) before and after stressing at 90 °C for 12 h in air (with 60% relative humidity) conditions under white light (1 sun equivalent). (B) DSC thermograms of both NDI-(PhBr)_2_ (blue) and NDI-(EtPA)_2_ (red).

Despite the evident stability of these films under common operating conditions, consideration must also be given to the morphological stability of a material. Previously, it has been reported that organic materials can undergo phase changes at elevated temperatures that can result in poor operational stability over long periods of time.^48^ Differential scanning calorimetry (DSC) can identify such thermal transitions through analysis of the heat flow as a sample is heated and cooled. Previous reports have shown that films which exhibit thermal transitions in their DSC scans at low temperatures are more prone to instability over long periods of time.^48^ Figure 3B displays the DSC thermograms for NDI-(EtPA)_2_ and NDI-(PhBr)_2_ molecules. Heating both NDI-(EtPA)_2_ and NDI-(PhBr)_2_ induces recrystallization or melting events at temperatures of 340 and 300 °C respectively. Importantly, no obvious thermal transitions are observed at the temperatures associated with device fabrication or operation as the most extreme temperature the films would be subjected to is 150 °C during the perovskite deposition step. The combined findings of our FTIR spectra post stressing and DSC analysis support our conclusion that the NDI films are both chemically and structurally stable under operating conditions and should exhibit consistent performance over extended periods of time.

### 2.4 Sensitivity of Organic Transport Layers to Processing Solvents

The sequential nature of perovskite device fabrication necessitates that NDI ETLs in n-i-p devices remain resistant to the processing solvents used in depositing subsequent layers, assuming those layers are deposited via spin-coating, the current standard in the field. Given that molecular thin films are often dominated by noncovalent interactions, processing solvents such as dimethylformamide (DMF) and dimethyl sulfoxide (DMSO) can disrupt intermolecular interactions, and hence solvent–film interactions should not be overlooked. Furthermore, the degree to which solvents interact with organic materials can often be impacted by the functional groups present. This behavior has been observed in NDIs whereby, for instance, functionalization with alkyl chains results in stronger solvent-NDI molecule interactions and potential dissolution.^49^ Therefore, it is possible that the differing nature of the ethyl phosphonic acid and aryl bromide functional groups would result in varying degrees of interaction with the processing solvents used.

To test the sensitivity of our NDI ETLs to subsequent steps of device fabrication, a solvent wash was conducted. In this process, which is shown schematically in Figure S40, we simulated the conditions present during perovskite deposition without any perovskite precursors to allow for the solvent - ETL interactions to be gauged without the formation of a perovskite phase. Initially, 90 μL of a 2:1 DMF:DMSO mixture was spin-coated onto the NDI films to replicate the perovskite deposition step. This was followed by spin-coating 250 μL of chlorobenzene, which acts as an antisolvent during perovskite deposition, before annealing at 150 °C for 10 min. Preliminary indication into the solvent sensitivity of our films was obtained through contact angle measurements, shown in Figure S41. Both film surfaces exhibit changes in the water contact angle on solvent washing, indicating some changes in surface polarity.

Synchrotron-based grazing incidence wide-angle X-ray scattering (GIWAXS) was utilized to further assess the impacts of solvent washing on the structure of our NDI films. Figure 4A shows the two-dimensional scattering patterns of NDI-(EtPA)_2_ films at an incident angle α = 0.5^°^ before and after washing. The unwashed NDI-(EtPA)_2_ sample exhibits a Debye–Scherrer ring at *q*_z_ = 2.0 Å^–1^, which is indicative of crystallinity. Increasing intensity around *q*_r_ = 0 Å^–1^ suggests that the material is also exhibiting some degree of preferential face-on orientation. We attribute this feature to ordering along the (112̅) plane, which has been previously attributed to π–π stacking of the NDI cores.^14^ Upon washing, the Debye–Scherrer ring disappears almost entirely for the NDI-(EtPA)_2_ film. This suggests that the washing process removes all crystalline features of the film. As NDI-(EtPA)_2_ is polar and protic, it is expected to undergo favorable interactions with DMSO and DMF. The observed sensitivity to processing solvents is therefore not unexpected. The two-dimensional scattering patterns of NDI-(PhBr)_2_ before and after washing are shown in Figure 4B. The unwashed NDI-(PhBr)_2_ sample exhibits a more complicated diffraction pattern, a characteristic that has been observed before in NDI based materials.^50,51^ A crystalline feature can be observed at *q*_z_ = 1.7 Å^–1^, which we attribute to stacking in the (010) plane, a feature that the literature reports is also due to π–π stacking.^52,53^ The more intense diffraction around *q*_r_ = 0 Å^–1^ implies preferential face-on orientation of the molecules. The presence of multiple features and an increase in amorphous background signal at higher q values point toward a film exhibiting crystallographic variation. This could help explain the tail and reduced vibronic features observed in the optical absorption of NDI-(PhBr)_2_ which we previously attributed to local disorder. Upon washing, the features at *q*_z_ = 1.7 Å^–1^ and *q*_z_ = 1.0 Å^–1^ disappear and a new broad Debye–Scherrer ring appears at *q*_z_ = 0.6 Å^–1^. This is evidence that while NDI-(PhBr)_2_ is recrystallizing into a different structure on washing, a greater portion of the film remains than in the instance of NDI-(EtPA)_2_.

**Figure 4:**
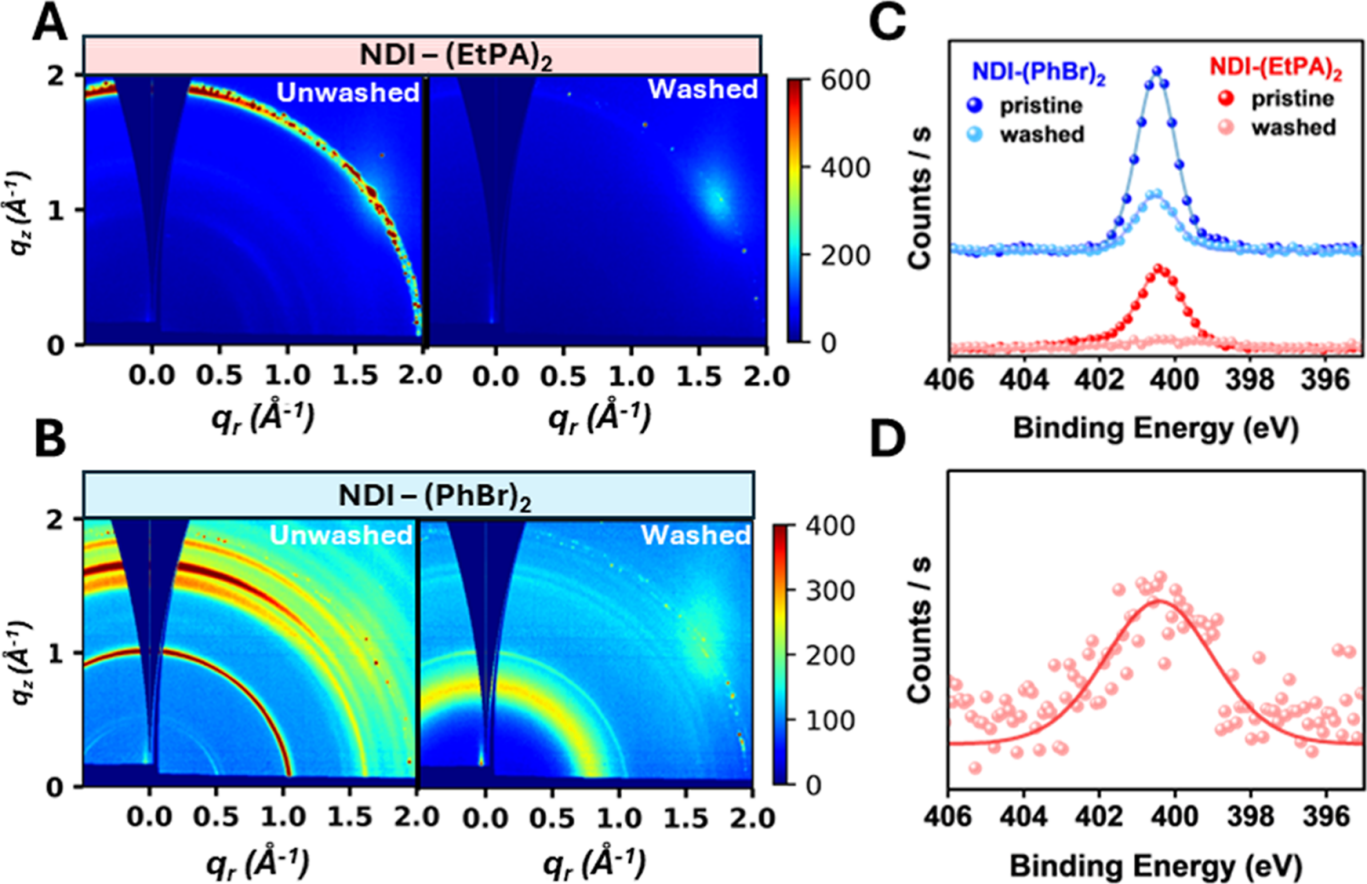
2D GIWAXS scattering pattern for (A) NDI-(EtPA)_2_ and (B) NDI-(PhBr)_2_ before (left) and after (right) solvent washing. (C) N 1s XPS of NDI-(EtPA)_2_ (red) and NDI-(PhBr)_2_ (blue) before and after solvent washing. (D) Rescaled N 1s XPS of NDI-(EtPA)_2_ after washing. The spheres represent the recorded data, while the lines represent the fitted envelope.

To help strengthen any conclusions made about the nature of the films remaining after the solvent wash, we conducted XPS on films of NDI-(PhBr)_2_ and NDI-(EtPA)_2_ before and after washing. The N 1s spectra for both films are shown in Figure 4C. While prominent N 1s signals are observed before washing for both films, significant reductions in signal intensity are observed after washing. For NDI-(PhBr)_2_, although a reduction in signal is observed, a significant N 1s signal remains after washing. This corroborates our GIWAXS findings and allows us to conclude definitively that while NDI-(PhBr)_2_ does experience interactions with processing solvents, a thin film remains upon washing, albeit with an altered crystal structure. The N 1s signal from NDI-(EtPA)_2_ is almost completely absent after solvent washing which corroborates the absence of crystalline features in the GIWAXS patterns. These findings suggest that the bulk portion of the NDI-(EtPA)_2_ film is being removed. Despite the large reduction in intensity, a small N 1s signal can still be observed in the XPS spectrum, as shown in Figure 4D. To ensure that this signal is not due to surface contamination, a P 2p spectrum is also shown in Figure S42. A clear P 2p peak is evident at 134.1 eV, which aligns with the peak observed in the unwashed sample. Another peak at 139.2 eV can also be observed in the spectrum. We assign this peak to the FTO given that this peak is observed in the XPS spectrum of bare FTO, shown in Figure S43.

### 2.5 Perovskite Solar Cell Performance

To evaluate the performance of these organic films as ETLs, n-i-p solar cells with the architecture FTO/NDI-ETL/Cs_0.09_FA_0.91_PbI_3_/PEAI/Spiro-OMeTAD/Au were fabricated, as shown schematically in Figure S44. Detailed information on the fabrication process can be found in the experimental section. The efficacy of the films as ETLs for photovoltaic application was evaluated by comparing *J*–*V* curves under illumination. The two champion *J*–*V* scans for NDI-(PhBr)_2_ and NDI-(EtPA)_2_ are displayed in Figure 5A. PCEs of 15.6% and 14.1% were achieved, respectively. To the best of our knowledge, these NDI containing devices outperform any previously reported PSCs utilizing thermally evaporated NDI ETLs. Figure 5B shows the distribution of PCEs for NDI-(EtPA)_2_, NDI-(PhBr)_2_ and our reference devices utilizing a TiO_2_ ETL. The distributions of the other key performance metrics: *V*_OC_, *J*_SC_, and fill factor (FF), which display similar trends to Figure 5B, and can be found in Figure S45. While both NDI-based devices exhibit more variance in their performance than the reference devices, NDI-(EtPA)_2_ exhibits considerably higher variance and, in general, lower efficiency than NDI-(PhBr)_2_. Given the similar band gaps and frontier orbital energies of these two molecules, these differences likely arise from morphological or structural differences between the NDI films. We hypothesize that the partial dissolution of the NDI-(EtPA)_2_ film on deposition of the perovskite layer, as shown by GIWAXS and XPS, results in inconsistent film coverage and hence lower performances and higher variances in general.

**Figure 5:**
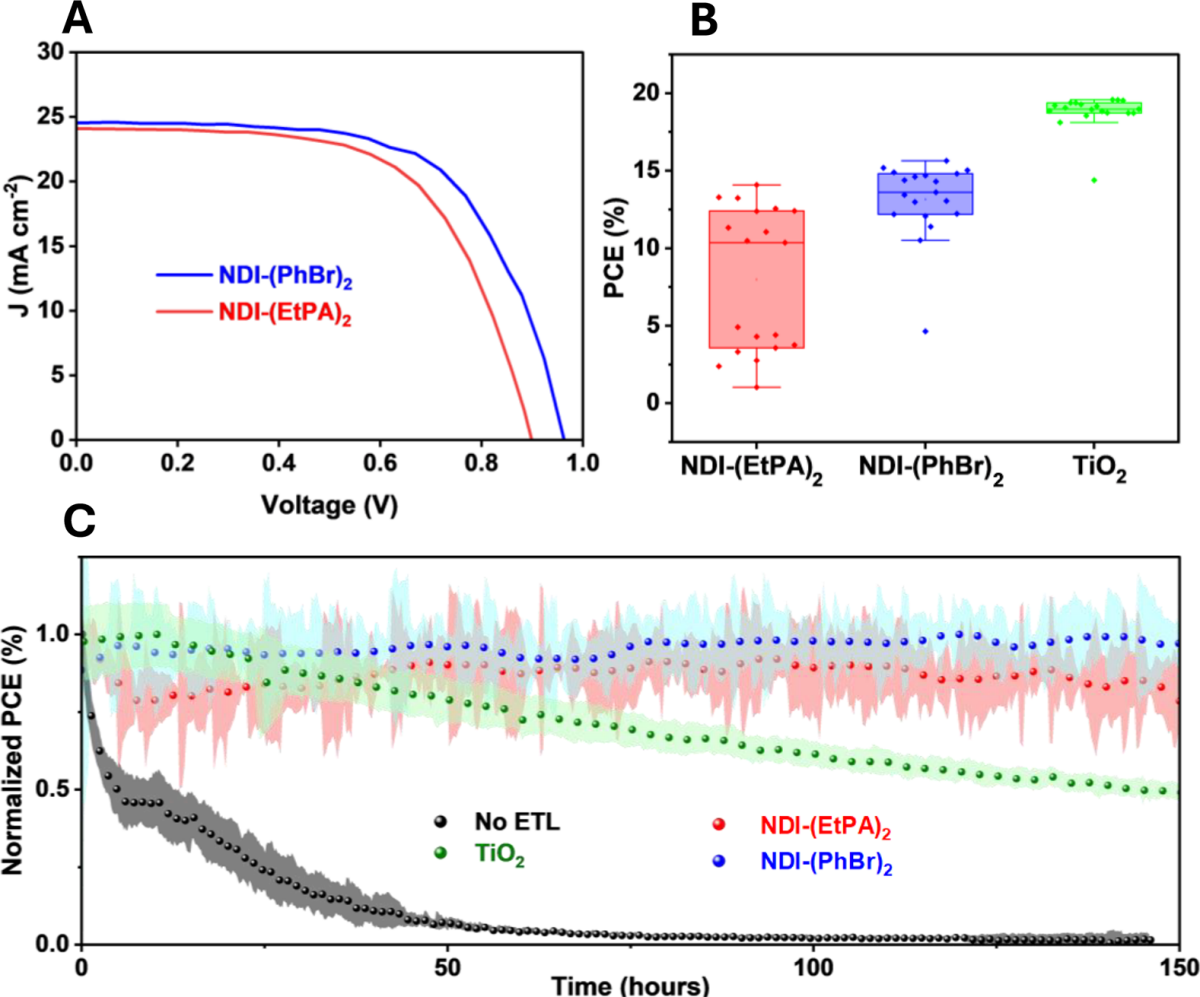
(A) Reverse *J*–*V* scan for both NDI-(EtPA)_2_ (red) and NDI-(PhBr)_2_ (blue). (B) PCE box plot for NDI-(EtPA)_2_ (red), NDI-(PhBr)_2_ (blue), and reference (green) devices. The solid points represent individual data points, rhe horizontal bar represents the median value of a distribution while the box signifies the interquartile range. (C) MPP tracking of NDI-(EtPA)_2_ (red), NDI-(PhBr)_2_ (blue), our TiO_2_ reference devices (green), and devices without an ETL (black) over a 200 h period at a temperature of 65 °C. The distribution shows the standard deviation associated with a particular time point.

To study the impact of long-term operation on our NDI transport layers we conducted a long-term device stability study. These stability measurements were conducted under 1 sun equivalent illumination at a temperature of 65 °C under a N_2_ atmosphere with tracking of the PCE. This data was smoothed using a Lowess algorithm to reduce high frequency noise while accurately representing the long-term trends. Normalized and raw data sets can be seen in Figures 5C and S46 respectively. Both devices containing NDI-(EtPA)_2_ and NDI-(PhBr)_2_ exhibit long-term stability over 150 h of operation, outperforming our TiO_2_ reference devices, despite the increased variability in the performance of our NDI-containing devices throughout the measurement. If we were to assume the NDI layer was removed completely on washing, we would expect an FTO/perovskite interface to be present in our device. As can be seen from Figure 5C, this interface lacks long-term stability in its performance, with device failure occurring within 50 h of operation. The considerable improvement in long-term performance for devices containing NDI-(EtPA)_2_ is evidence of a monolayer remaining that aids in the extraction of electrons and stabilization of the perovskite/FTO interface.

## 3 Conclusions

This work introduces a family of NDI molecules, two of which were shown to act as effective electron transport layers in perovskite solar cells. It was found that ethyl phosphonic acid groups have a lower temperature threshold for thermal decomposition, which complicates their thermal evaporation. FTIR and XPS showed that the molecular structure of NDI-(PhBr)_2_ is consistent between thermally evaporated, and solution processed films while NDI-(EtPA)_2_ undergoes chemical changes on evaporation. Despite these chemical changes, it was shown that NDI cores are present in both thermally evaporated films. Optoelectronic properties were probed, and it was found that the thermally evaporated NDI thin films possessed ideal large bandgaps. We demonstrated that while both NDI-(EtPA)_2_ and NDI-(PhBr)_2_ thin films are sensitive to processing solvents used for perovskite deposition, the layers are not completely removed during the deposition process and still preserve their electron transport properties and charge selectivity. This enabled devices to be fabricated using thin films of both NDI-(EtPA)_2_ and NDI-(PhBr)_2_ with efficiencies of 14.1% and 15.6%, respectively. Both NDI-derivatives resulted in devices that outperform previous attempts at thermally evaporated NDI ETLs. Finally, we demonstrated the thermal and structural stability of our ETLs at operating conditions, and fabricated devices that demonstrate outstanding stability over 150 h of continuous operation. This work not only establishes new efficiency records for thermally evaporated NDI thin films in ETL applications but also identifies key synthetic considerations that are required for the design of scalable organic ETLs in the future.

## 4 Experimental Section

### 4.1 Molecule Synthesis

Molecules were synthesized via the methods outlined in the synthesis section of the Supporting Information.

### 4.2 Substrate Cleaning

1 in. × 1 in. substrates (glass, FTO, FTO patterned glass) were cleaned via sequential sonication for 15 min in 2% mucasol solution, distilled water, acetone (Sigma-Aldrich, ≥99.5%), and finally isopropyl alcohol (Fischer Chemical). PET-ITO underwent a similar cleaning procedure with the acetone wash omitted. After cleaning, the substrates were dried using a nitrogen gun. Prior to deposition of subsequent layers, substrates were placed in a UV–ozone cleaner for 15 min.

### 4.3 NDI Evaporation

NDI layers were deposited via thermal evaporation using a Kurt J. Lesker MiniSpectros series low-temperature evaporator. Substrates were placed in the evaporator and the system was pumped down to at least 1 × 10^–5^ Torr. The substrates were rotated at a rate of 10 rpm during the deposition procedure to ensure uniformity. Specific weights of the molecules were weighed out (≈3 mg, ≈30 mg, ≈100 mg) in a nitrogen atmosphere before being placed in the evaporator. The molecules were heated gently until deposition was recorded via a 6 MHz gold Quartz Crystal Microbalance (QCM) positioned close to the thermal source. The deposition was continued until no more rate was observed despite increasing the source temperature.

### 4.4 Thin Film and Molecular Characterization

^**1**^ **H Nuclear Magnetic Resonance (NMR)** spectra for all molecular precursors were acquired through BrukerAvance IIIHD 500 MHz or Bruker Avance IIIHD 700 MHz instruments using either CDCl_3_ or DMSO – *d6* as a solvent; the residual CHCl_3_ peak was used as a reference for all reported chemical shifts (1H: δ = 7.26 ppm, 13C: δ = 77.16 ppm) and for DMSO-*D6* (^1^H: δ = 2.50 ppm, ^13^C: δ = 39.48–40 ppm). **Differential Scanning Calorimetry (DSC)** was performed using a TA Instruments Q200 with heating and cooling rates of 10 °C min^–1^. Powder samples with a mass of approximately 5 mg were used and encapsulated in closed DSC aluminum pans under a controlled N_2_ atmosphere. **Thermogravimetric Analysis (TGA)** was performed using a Mettler Toledo TGA2 STAR System Thermogravimetric Analyzer. Five mg of precursor powders were heated to a temperature of 700 °C at a temperature ramp rate of 15 °C min^–1^ in an inert atmosphere of 15 mL min^–1^ of nitrogen. **Fourier Transform Infrared Spectroscopy (FTIR)** was measured using a Nicolet 6700 FT-IR. FTIR spectra were measured via attenuated total reflectance (ATR) and to ensure close contact between the sample and the diamond, films were deposited on flexible substrates of commercially available PET-ITO. Each FTIR scan was repeated 64 times. **X-ray Photoelectron Spectroscopy (XPS):** XPS was conducted using a Thermo NEXSA G2 system using a monochromatic Al Kα X-ray source (1486.6 eV). Survey scans were acquired through averaging two measurements with a 200 eV pass energy and 1 eV energy step size. Elemental scans were acquired using 50 eV pass energy and 0.1 eV energy step size. Elemental scans were recorded for all elements expected in the NDI molecules. Elemental composition analysis was performed using the Thermo Scientific Avantage data system for surface analysis. **Ultraviolet-Photoelectron-Spectroscopy (UPS)** was also conducted on a Thermo NEXSA G2 system. A He 1 source (21.22 eV) of UV light was used and a 5 V bias was placed on the system. Conductive FTO substrates were used to ensure electronic connection between the samples and the spectrometer. Onset values were calculated via extrapolation of initial electron onsets to the baseline. **Optical absorption spectra** were taken with a Jasco V-630 spectrometer. **Spectroscopic** e**llipsometry** measurements were conducted using a Woollam M-2000 ellipsometer. Transmittance measurements were used to estimate film thickness. For silicon substrates, Cauchy and BSPline models were fitted to estimate thickness. **Contact Angle Goniometry** measurements were taken using ramé-hart Goniometer/Tensiometer model 290 and analyzed through ImageJ software via low bond axisymmetric drop shape analysis. To evaluate the surface energy, 2.5 μL of diiodomethane (nonpolar) and deionized water (polar) were deposited individually onto the surface of interest. **Grazing Incidence Wide Angle X-ray Scattering (GIWAXS)** measurements were performed at beamline 11-BM at National Synchrotron Light Source II in Brookhaven National Laboratory. The X-ray beam energy was 13.5 keV with a spot size of 0.2 mm × 0.05 mm. The samples were irradiated for 10 s with an incident angle of 0.5°. Beam divergence was 1 mrad with an energy resolution of 0.7%. The data were analyzed using the SciAnalysis package provided by the beamline.

### 4.5 Device Fabrication

A compact layer of TiO_2_ (c-TiO_2_) was deposited via spray pyrolysis, using a solution comprising 720 μL of titanium di-isopropoxide bis­(acetylacetonate) (75% in 2-propanol, Sigma-Aldrich), and 10.8 mL of 99.9% pure anhydrous ethanol (SigmaAldrich). A 3.0 L min^–1^ flow of oxygen was used as carrier gas to spray the solution. Spraying was conducted with a Sparmax spray gun. The prepared solution was sprayed onto substrates preheated at 450 °C. Twenty second spray cycles were performed with a 30 s delay between each cycle. Spray cycles were continued until no solution remained. The substrates were then annealed at 450 °C for another 30 min postdeposition. Substrates were then allowed to cool to room temperature before the mesoporous TiO_2_ layer (TiO_2_-mp) was deposited via static spin-coating. A 150 mg mL^–1^ solution of TiO_2_ paste (30 nm nanoparticles, GreatSolar) in ethanol (99.9% pure, anhydrous, Sigma-Aldrich) was used. The spin-coating parameters were 2000 rpm for 10 s with an acceleration rate of 2000 rpm s^–1^. The deposited substrates were immediately moved to a hot plate and heated at 100 °C for 10 min. The mesoporous films were subsequently sintered by heating of the substrates at 450 °C and maintaining the temperature for 30 min. Between deposition of subsequent layers, substrates were kept in an inert nitrogen atmosphere with concentrations of O_2_ and H_2_0 < 10 ppm. To study the molecular ETLs deposited by evaporation, as described above, we substituted the TiO_2_ compact and mesoporous layers with NDI thin films. We aimed for films with a thickness of around 30 nm.

The perovskite layer was deposited via spin coating of a 1.2 M solution of Cs_0.09_FA_0.91_PbI_3_. Precursor powders of PbI_2_ (Tokyo Chemical Industry, >98%), formamidinium iodide (FAI, Dynamo) and cesium iodide (CsI, Sigma-Aldrich) were weighed inside a glovebox with a 5% stoichiometric excess of PbI_2_. Precursor powders were dissolved in a 2:1 volume ratio of *N*,*N*-dimethylformamide (DMF, Acros Organics, ≥99.8%)/dimethyl sulfoxide (Acros Organics,>99.8%). A two-step spin-coating process was used. First, 90 μL was deposited on a static substrate before being spin-coated at 1000 rpm for 10 s and then 6000 rpm for 20 s. Twenty-six s into the spin coating procedure, 250 μL of chlorobenzene (Sigma-Aldrich, anhydrous) was dropped onto the substrate. Substrates were then annealed at 150 °C for 10 min. 90 μL of phenethylammonium iodide (PEAI, Dynamo) solution with a concentration of 1 mg mL^–1^ in IPA (Sigma-Aldrich, 99.9%) was used as a surface treatment. The spin coating recipe used was 20 s of spinning at 5000 rpm with an acceleration rate of 5000 rpm s^–1^. After PEAI deposition, the substrate was annealed at a temperature of 100 °C for 10 min.

A doped Spiro-OMeTAD solution was prepared for deposition of the hole transport layer. 100 mg of Spiro-OMeTAD (1-Material) was dissolved in 1098.38 μL of chlorobenzene (Acros Organics, 99.9%) to form a 0.07 M solution. 18.13 μL of 1.8 M lithiumbis­(trifluoromethane)­sulfonimide (Li-TFSI, Sigma-Aldrich) in acetonitrile (Sigma-Aldrich, anhydrous, 99.8%), 39.45 μL of 4-tertbutylpyridine (tBP, Sigma-Aldrich, 98%) and 9.79 μL of 0.25 M tris­(2-(1H-pyrazol-1-yl)-4-*tert*-butylpyridine)­cobalt­(III) tri­[bis­(trifluoromethane)­sulfonimide] (FK 209 Co (III), Sigma-Aldrich) in acetonitrile were added as dopants. 90 μL of doped Spiro-OMeTAD solution was subsequently spin coated dynamically with 3000 rpm for 30 s with an acceleration of 3000 rpm s^–1^.

The edges of the substrates were then cleaned with DMF and acetonitrile to remove the perovskite and hole transport layers in ambient air. 50 nm of gold (KJLC, >99.999%) was deposited via thermal evaporation as a back contact. Shadow masks were used to define 8 pixels per substrate, each with an active area of 0.128 cm^2^.

### 4.6 Device Characterization

The photovoltaic performance of devices was evaluated using a Fluxim Litos Lite setup, equipped with a Wavelabs Sinus-70 AAA solar simulator with AM1.5 spectrum for excitation. The current–voltage (*J*–*V*) characteristics were acquired with forward and reverse scans ranging from −0.5 to 1.2 V with a scan rate of 50 mV s^–1^. The stabilized power output was acquired using an MPP tracking algorithm for 120 s. Devices were not preconditioned before measurement. Masking was used during the measurement, defining a pixel area of 0.0625 cm^2^. Nitrogen was blown in the measurement chamber during characterization. No temperature control was applied.

For the long-term thermal stability measurement, a Fluxim Litos, a stress-test platform for degradation analysis, was employed. The solar cells were stressed at temperature of 65 °C in N_2_ rich environment, under 1 sun equivalent illumination without UV light and with continuous MPP tracking. During the stability measurement, automated acquisition of *J*–*V* scans in both reverse and forward directions were taken every 12 h. Data smoothing was performed using the Lowess (locally weighted scatterplot smoothing) algorithm in OriginPro, with a window spanning the nearest 506 neighboring data points for the stability data in Figure 5c due to some degree of variability in the measurement.

## Supplementary Material



## Notes

Synthetic information and corresponding NMR data for all synthesized molecules is provided. Additional Supporting Information includes supplementary figures with experimental details, characterization data, and device performance metrics (PDF)
