# Supplementary material for: Thermally Evaporated Naphthalene Diimides as Electron Transport Layers for Perovskite Solar Cells
Source: Chem Mater. 2025 Aug 26;37(17):6655–66. doi: 10.1021/acs.chemmater.5c01186 (PMC12424226; doi:10.1021/acs.chemmater.5c01186)
Supplement: Supplementary file 1 [file cm5c01186_si_001.pdf]

Supporting Information

Thermally Evaporated Naphthalene Diimides as Electron Transport Layers for Perovskite Solar Cells

Jack Lawton[1], Justine S. Wagner[2], Xiangyu Xiao[1], Sanggyun Kim[1], Anna M. Österholm[2], D. Eric Shen[2], Sina Sabury[2], Carlo A. R. Perini[1], Kunal Datta[1], Diana K. LaFollette[1], Ruipeng Li[3], John R. Reynolds[1, 2], Juan-Pablo Correa-Baena[1, 2*]

[1]School of Materials Science and Engineering, Georgia Institute of Technology, North Ave NW, Atlanta, Georgia 30332, USA

[2]Georgia Institute of Technology School of Chemistry and Biochemistry, Georgia Institute of Technology, Atlanta, Georgia 30332, USA

[3]National Synchrotron Light Source II, Brookhaven National Laboratory, Upton, New York 11973, United States of America

Corresponding author: JPCB jpcorrea@gatech.edu

***Side Chain Synthesis*:**

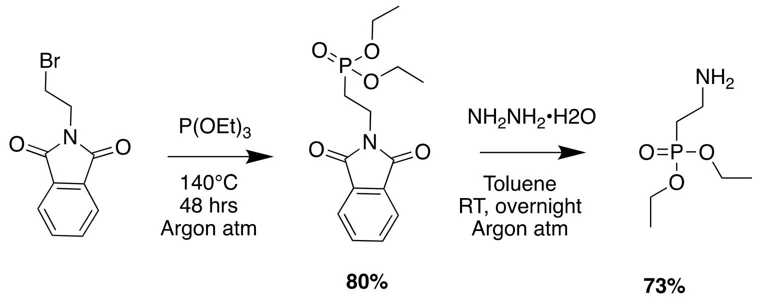

Supplementary Figure 1: Synthesis of Diethyl P-(2-aminoethyl)phosphonate

Diethyl (2-(1,3-dioxoisoindolin-2-yl)ethyl)phosphonate:

A slightly modified synthetic protocol[1] was followed: to a 100 mL round bottom flask charged with a magnetic stir bar N-(2-bromoethyl)phthalimide (3.6 g, 14.2 mmol, 1 eq.) and triethyl phosphite (12 mL, 70.8 mmol, 5 eq.) were added under an inert atmosphere. The vessel was then put onto a hot plate at 140 °C and stirred for 48 hours. Afterwards the reaction was cooled to room temperature and excess triethyl phosphite was distilled off. The crude was purified by silica gel chromatography using 100% DCM to 100% ethyl acetate which a viscous yellow oil was obtained (3.52 g, 80% yield). ^{1}H NMR (500 MHz, $CDCl_3$) δ 7.87 (dd, J = 5.5, 3.0 Hz, 2H), 7.74 (dd, J = 5.5, 3.0 Hz, 2H), 4.24 – 4.05 (m, 4H), 4.05 – 3.82 (m, 2H), 2.36 – 2.10 (m, 2H), 1.32 (t, J = 7.1 Hz, 6H). ^{31}P NMR (202 MHz, $CDCl_3$) δ 27.39. ^{13}C NMR (126 MHz, $CDCl_3$) δ 167.82, 134.04, 132.08, 123.32, 61.94, 61.88, 32.26, 32.24, 25.34, 24.23, 16.37, 16.32.

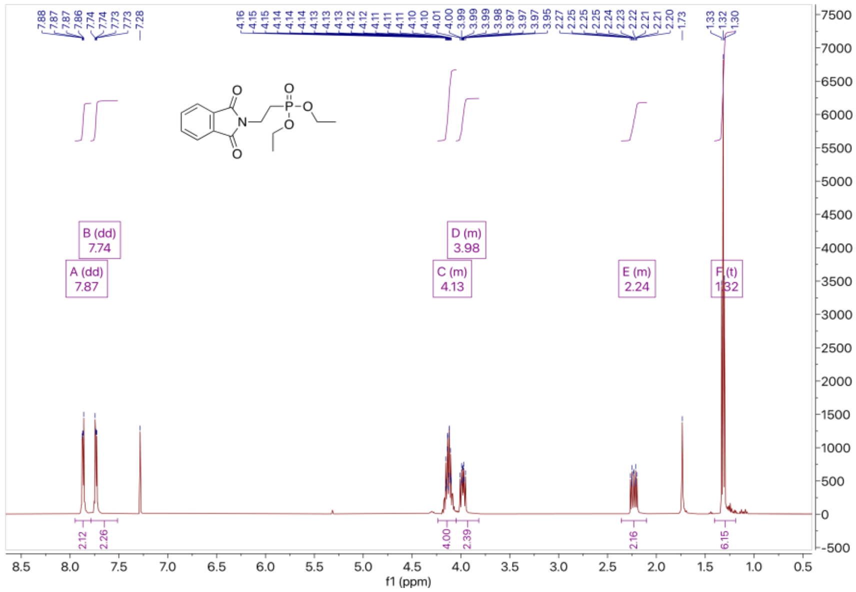

Supplementary Figure 2: ^{1}H NMR spectrum of diethyl (2-(1,3-dioxoisoindolin-2-yl)ethyl)phosphonate.

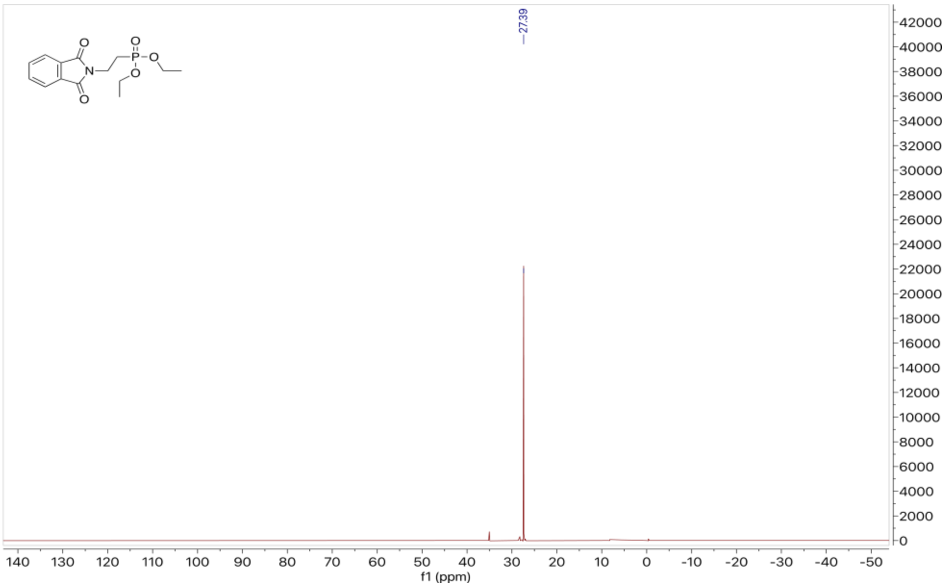

Supplementary Figure 3: ^{31}P NMR spectrum of diethyl (2-(1,3-dioxoisoindolin-2-yl)ethyl)phosphonate.

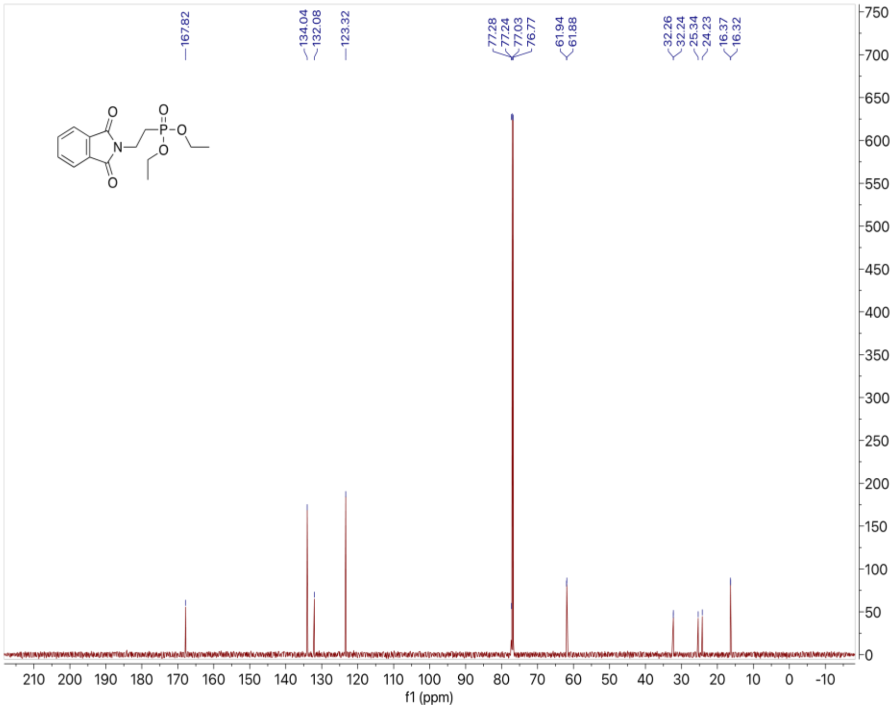

Supplementary Figure 4: ^{13}C NMR spectrum of diethyl (2-(1,3-dioxoisoindolin-2-yl)ethyl)phosphonate.

Diethyl P-(2-aminoethyl)phosphonate:

A slightly modified synthetic protocol[1] was followed: To a 250 mL round bottom flask charged with a magnetic stir bar diethyl (2-(1,3-dioxoisoindolin-2-yl)ethyl)phosphonate (3.52 g, 11.3 mmol, 1 eq.) and 50 mL of anhydrous toluene were added under an inert atmosphere. The solution stirred for 10 minutes at room temperature before a solution of hydrazine monohydrate (7 mL, 135.6 mmol, 12 eq.) was added and the reaction was left to stir overnight at room temperature. The reaction was then filtered, and the precipitate was washed excessively with ethyl acetate. All of the filtrate was collected and then concentrated in vacuo to yield a pale-yellow oil that was used without any further purification (1.5 g, 73% yield). ^{1}H NMR (500 MHz, $CDCl_3$) δ 4.29 – 3.82 (m, 4H), 2.95 (dt, J = 17.1, 7.0 Hz, 2H), 1.87 (dt, J = 17.9, 7.0 Hz, 2H), 1.27 (t, J = 7.1 Hz, 6H). ^{31}P NMR (202 MHz, $CDCl_3$) δ 30.86. ^{13}C NMR (126 MHz, $CDCl_3$) δ 61.49, 61.44, 36.33, 36.29, 30.58, 29.48, 16.43, 16.38.

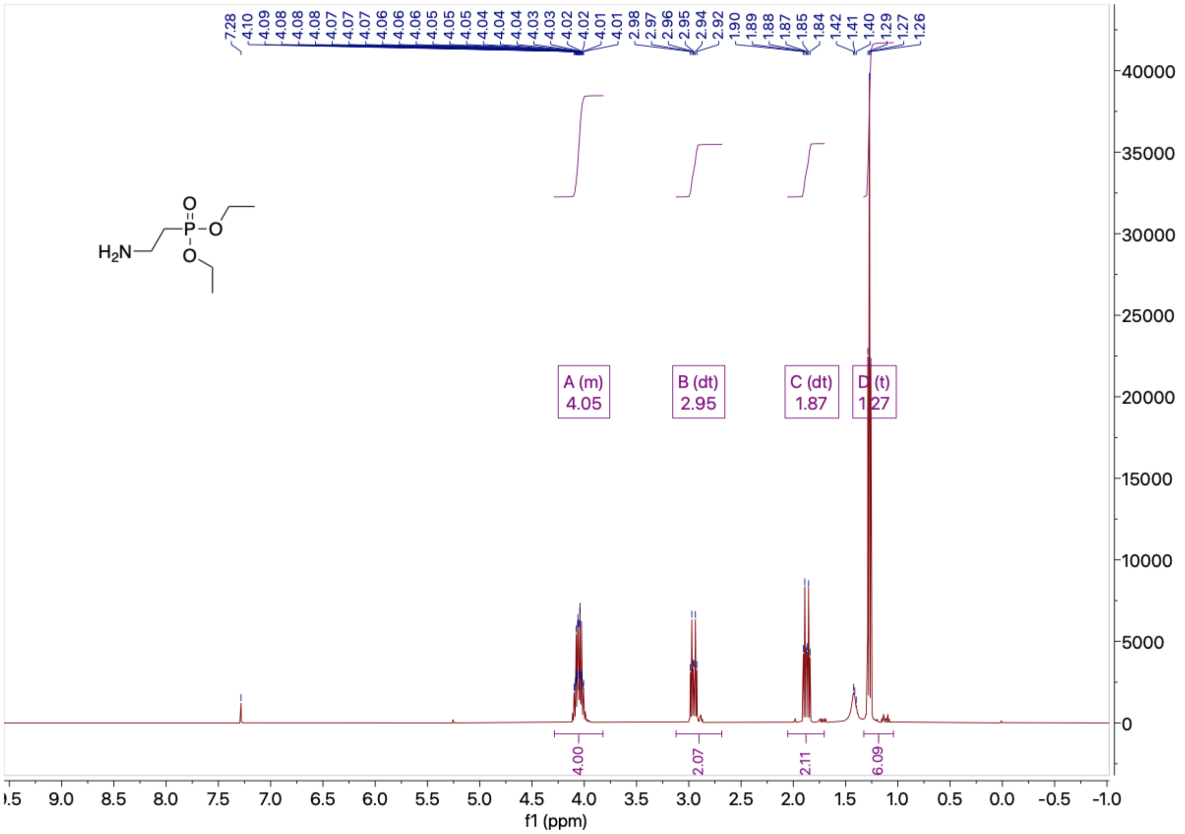

Supplementary Figure 5: ^{1}H NMR spectrum of diethyl P-(2-aminoethyl)phosphonate

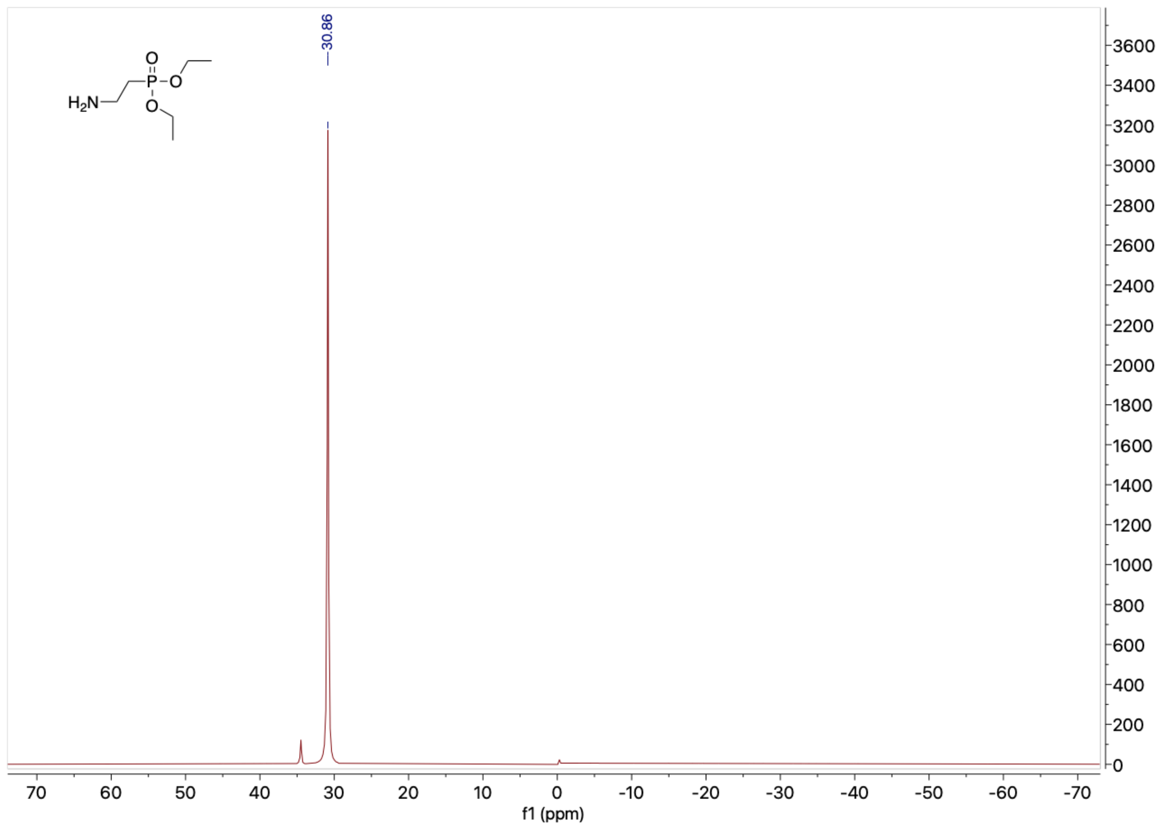

Supplementary Figure 6: ^{31}P NMR spectrum of diethyl P-(2-aminoethyl)phosphonate.

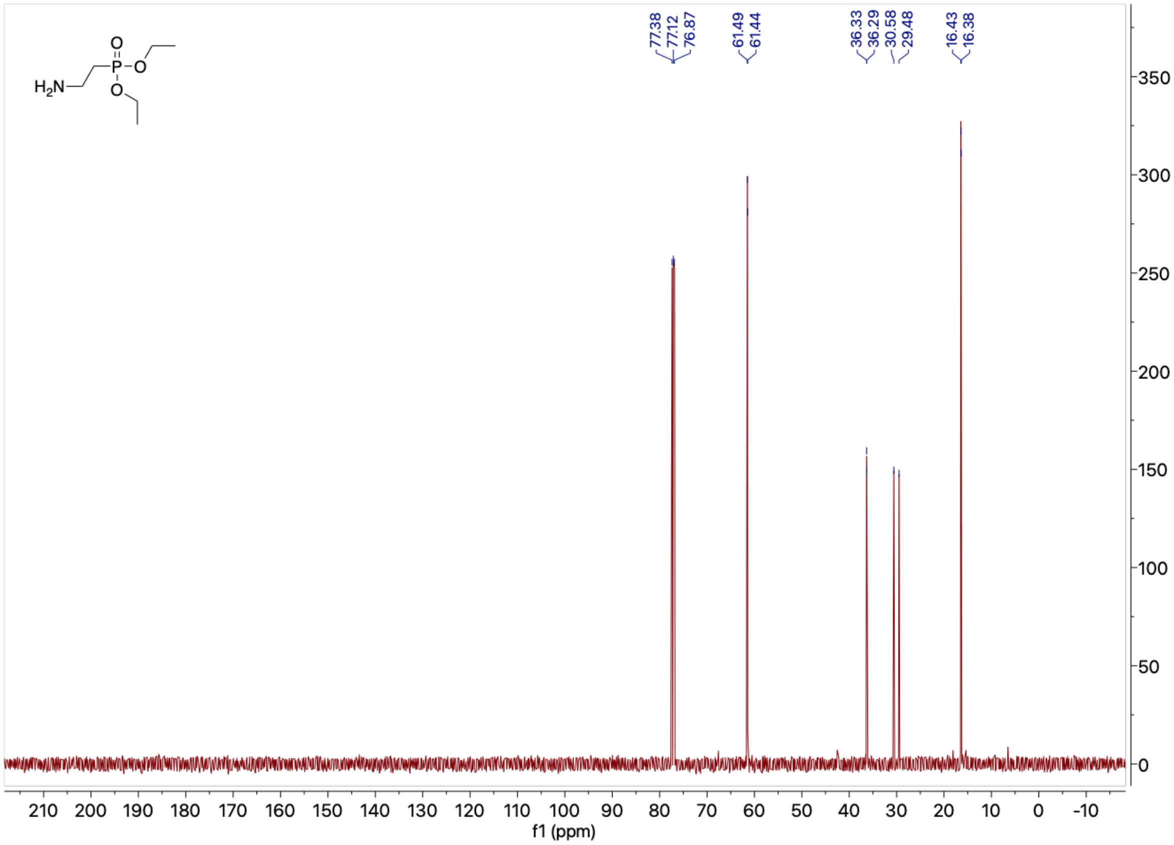

Supplementary Figure 7: ^{13}C NMR spectrum of diethyl P-(2-aminoethyl)phosphonate.

Compound Synthesis:

NDI-(EtPA)$_2$ precursor: Tetraethyl ((1,3,6,8-tetraoxo-1,3,6,8-tetrahydrobenzo[*lmn*][3,8]phenanthroline-2,7-diyl)bis(ethane-2,1-diyl))bis(phosphonate)

To a 250 mL round bottom flask charged with a magnetic stir bar, napthalenetetracarboxylic dianhydride (0.73 g, 2.7 mmol, 1 eq.) and 25 mL of glacial acetic acid were added. The reaction stirred for 10 minutes at 60 °C before diethyl P-(2-aminoethyl)phosphonate (1.5 g, 8.2 mmol, 3 eq.) was added. The temperature was raised to 90 °C and the reaction was left to stir for 48 hours. Afterwards, the reaction cooled to room temperature before precipitating into cold distilled water. The precipitate was filtered and washed excessively with distilled water. The filtered powder was then placed under high vacuum to dry for 48 hours to obtain a light brown powder (1.13 g, 69.7% yield). ^{1}H NMR (500 MHz, $CDCl_3$) δ 8.79 (s, 4H), 4.59 – 4.42 (m, 4H), 4.30 – 3.93 (m, 8H), 2.44 – 2.16 (m, 4H), 1.37 (t, J = 7.1 Hz, 12H). ^{31}P NMR (202 MHz, $CDCl_3$) δ 27.35. ^{13}C NMR (126 MHz, $CDCl_3$) δ 162.50, 131.11, 126.78, 126.60, 6.01, 61.96, 35.15, 24.94, 23.84, 16.47, 16.43.

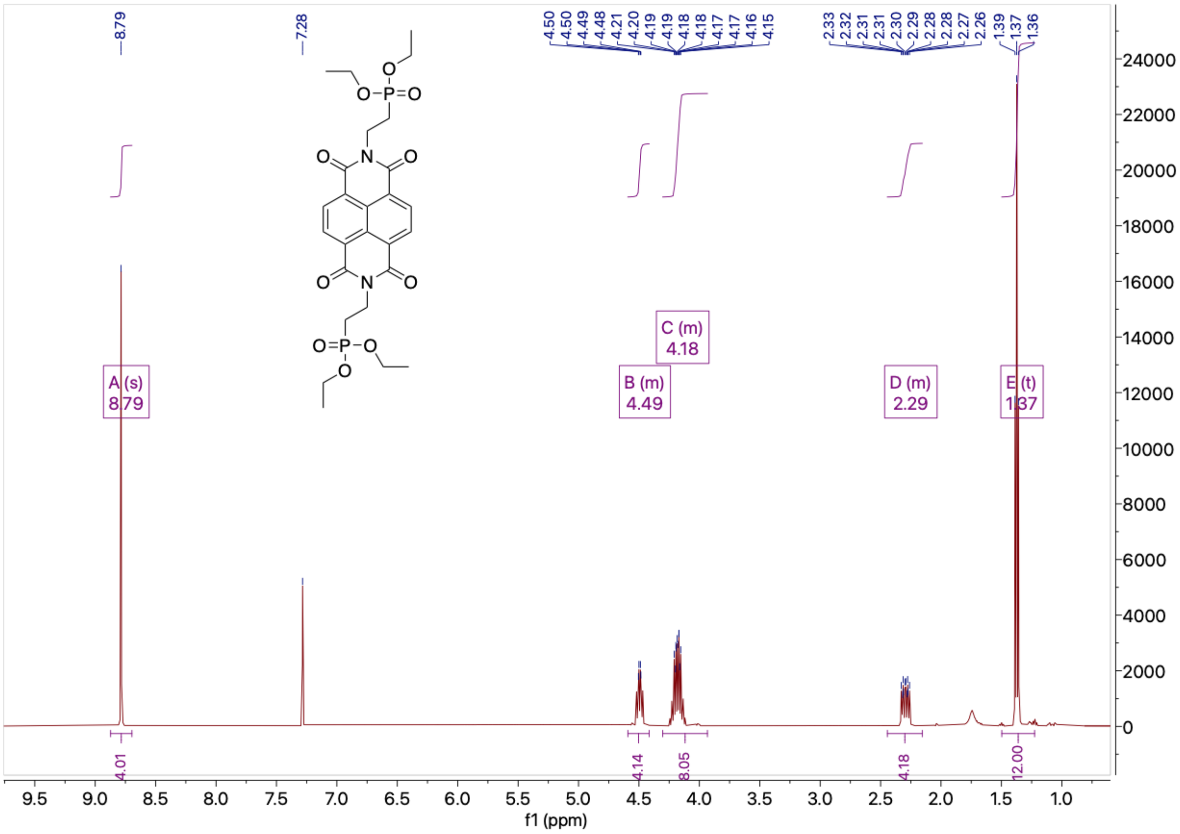

Supplementary Figure 8: ^{1}H NMR spectrum of tetraethyl ((1,3,6,8-tetraoxo-1,3,6,8-tetrahydrobenzo[*lmn*] [3,8]phenanthroline-2,7-diyl)bis(ethane-2,1-diyl))bis(phosphonate).

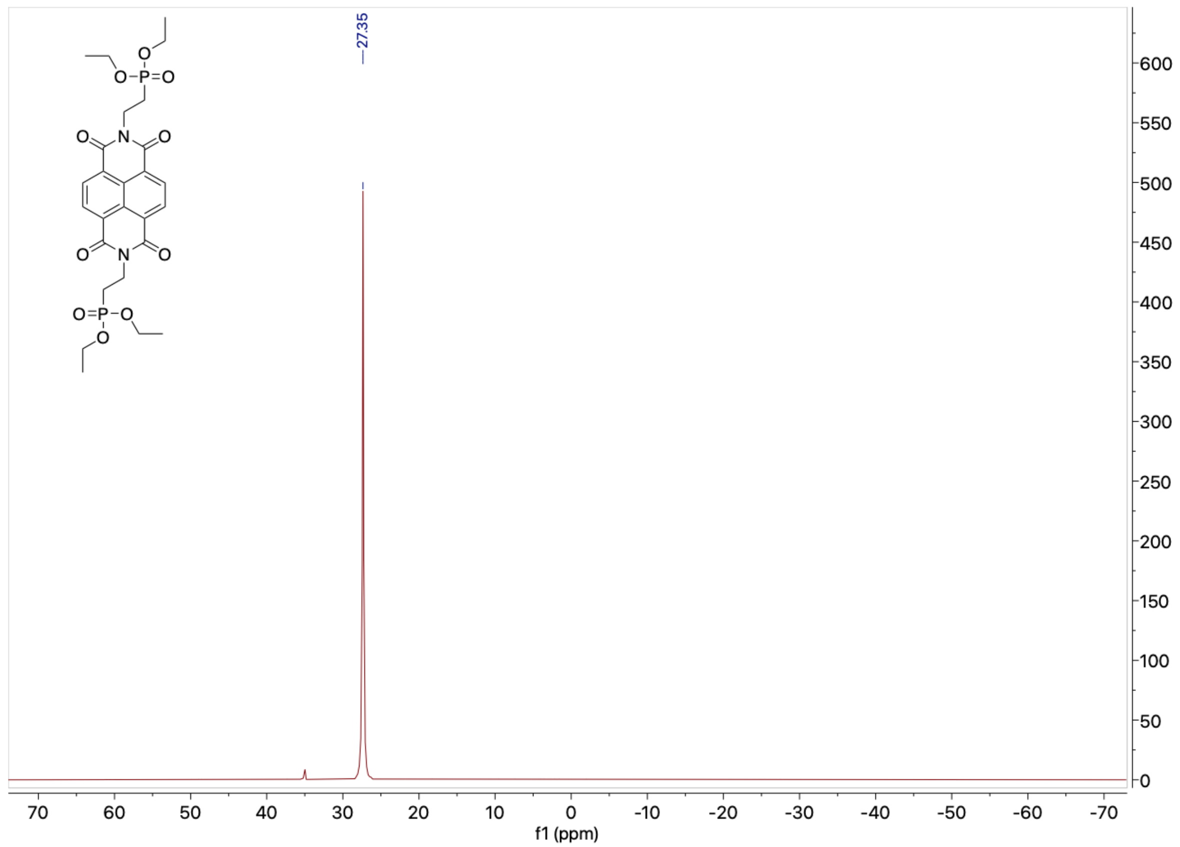

Supplementary Figure 9: ^{31}P NMR spectrum of tetraethyl ((1,3,6,8-tetraoxo-1,3,6,8-tetrahydrobenzo[*lmn*] [3,8]phenanthroline-2,7-diyl)bis(ethane-2,1-diyl))bis(phosphonate).

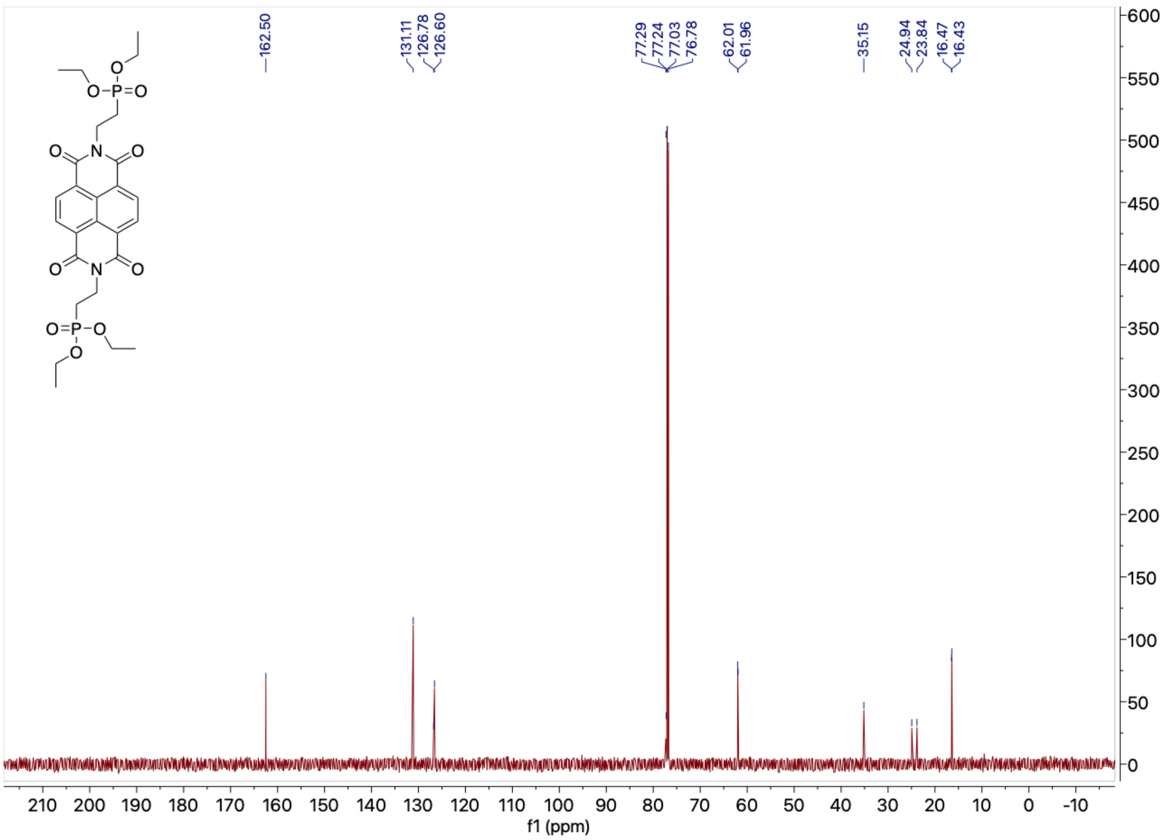

Supplementary Figure 10: ^{13}C NMR spectrum of tetraethyl ((1,3,6,8-tetraoxo-1,3,6,8-tetrahydrobenzo[*lmn*] [3,8]phenanthroline-2,7-diyl)bis(ethane-2,1-diyl))bis(phosphonate)

NDI-(EtPA)$_2$: ((1,3,6,8-tetraoxo-1,3,6,8-tetrahydrobenzo[lmn][3,8]phenanthroline-2,7-diyl)bis(ethane-2,1-diyl))bis(phosphonic acid):

To a 100 mL round bottom flask charged with a magnetic stir bar tetraethyl((1,3,6,8-tetraoxo-1,3,6,8-tetrahydrobenzo[*lmn*][3,8]phenanthroline-2,7-diyl)bis(ethane-2,1-diyl))bis(phosphonate) (1.13 g, 1.9 mmol, 1 eq.) and 25 mL of anhydrous dichloromethane were added under an inert atmosphere. After stirring for 10 minutes at room temperature, bromotrimethylsilane (2.9 g, 19.0 mmoles, 10 eq.) was added and the reaction was left to stir overnight at room temperature. After 12 hours, 3 mL of methanol was added and left to stir for an additional 3 hours. The solution was then concentrated to complete dryness and the powder was suspended in methanol and filtered. The product was then dried under high vacuum to receive a white powder (0.74 g, 81% yield). ^{1}H NMR (500 MHz, DMSO) δ 8.61 (s, 4H), 4.45 (t, J = 7.2 Hz, 4H), 4.25 (dt, J = 11.7, 6.8 Hz, 4H), 2.13 – 1.81 (m, 4H). ^{31}P NMR (202 MHz, DMSO) δ 22.15. ^{13}C NMR (126 MHz, DMSO) δ 162.90, 159.92, 142.49, 132.14, 130.81, 128.86, 126.88, 126.61, 125.10, 115.18, 35.95, 35.10, 27.51, 26.45.

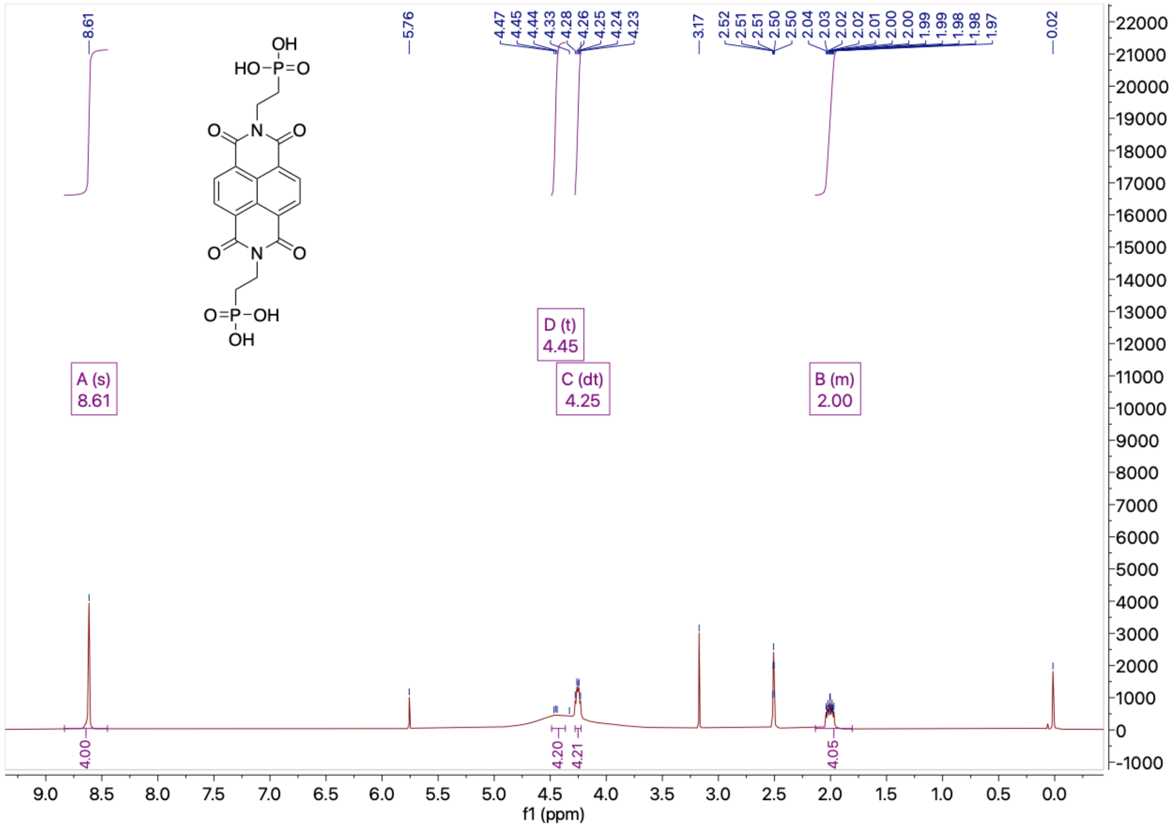

Supplementary Figure 11: ^{1}H NMR spectrum ((1,3,6,8-tetraoxo-1,3,6,8-tetrahydrobenzo[lmn][3,8]phenanthroline-2,7-diyl)bis(ethane-2,1-diyl))bis(phosphonic acid)

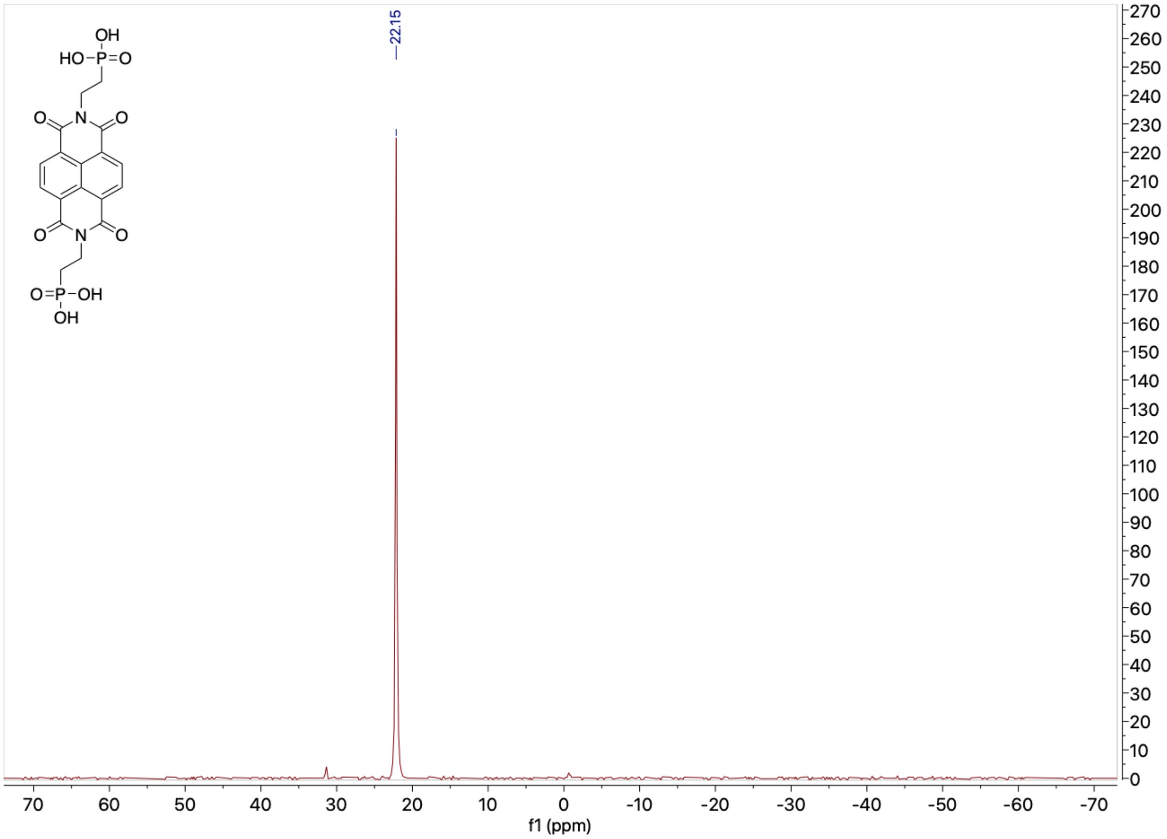

Supplementary Figure 12: ^{31}P NMR spectrum of ((1,3,6,8-tetraoxo-1,3,6,8-tetrahydrobenzo[lmn][3,8]phenanthroline-2,7-diyl)bis(ethane-2,1-diyl))bis(phosphonic acid)

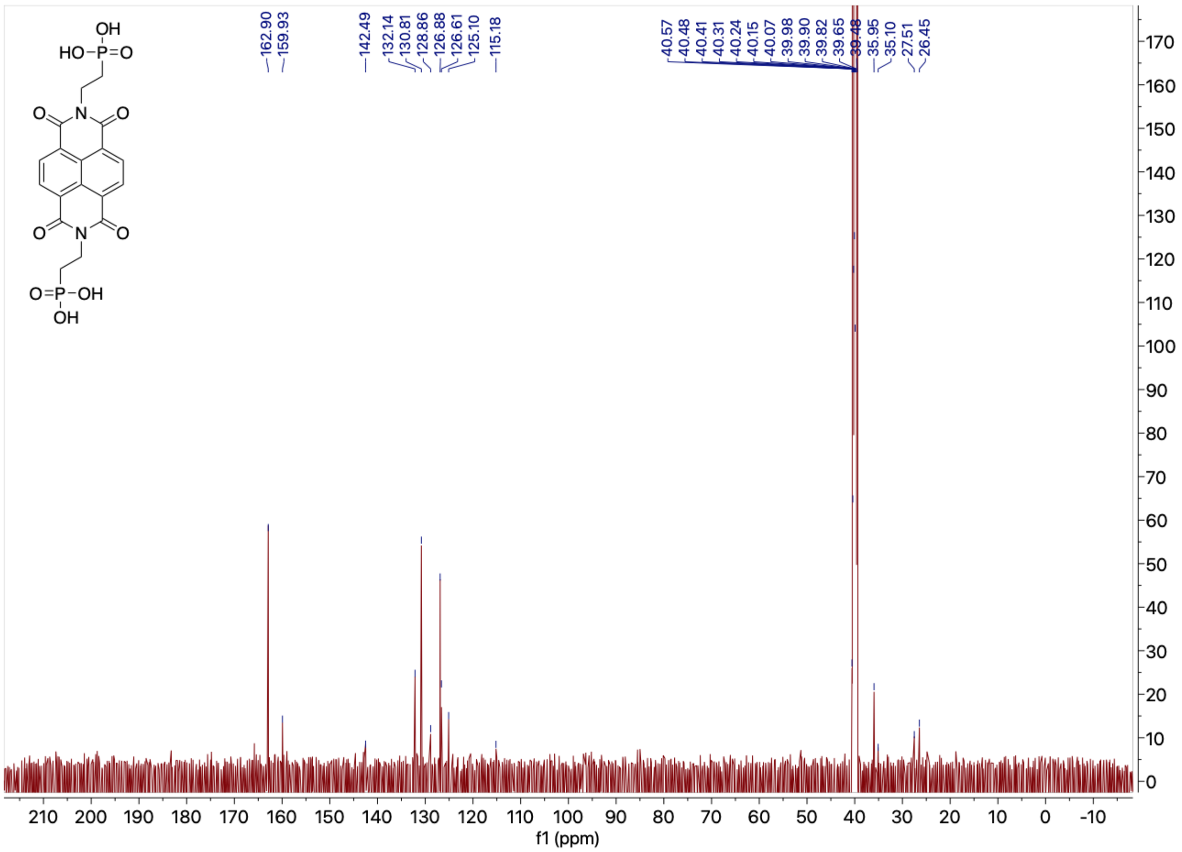

Supplementary Figure 13: ^{13}C NMR spectrum of ((1,3,6,8-tetraoxo-1,3,6,8-tetrahydrobenzo[lmn][3,8]phenanthroline-2,7-diyl)bis(ethane-2,1-diyl))bis(phosphonic acid)

NDI-(PhBr)$_2$: 2,7-bis(4-bromphenyl)benzo[lmn][3,8]phenanthroline-1,3,6,8(2H,7H)-tetraone:

To a round bottom flask charged with a stir bar napthalenetetracarboxylic dianhydride (6.0 g, 22.4 mmol, 1 eq.) and 4-bromoaniline (9.6 g, 55.9 mmol, 2.5 eq.) were added into 100 mL of anhydrous dimethylformamide. The reaction mixture was placed on a hot plate at 120 °C and left to stir overnight. Afterwards, the reaction cooled to room temperature, was filtered, and washed with methanol to obtain a yellow powder. ^{1}H NMR (500 MHz, DMSO) δ 8.74 (s, 4H), 7.79 (d, J = 8.5 Hz, 4H), 7.46 (d, J = 8.5 Hz, 4H). ^{13}C could not be obtained due to limited solubility and low resolution. ESI-MS: m/z theoretical 574.9236586, obtained 574.9240.

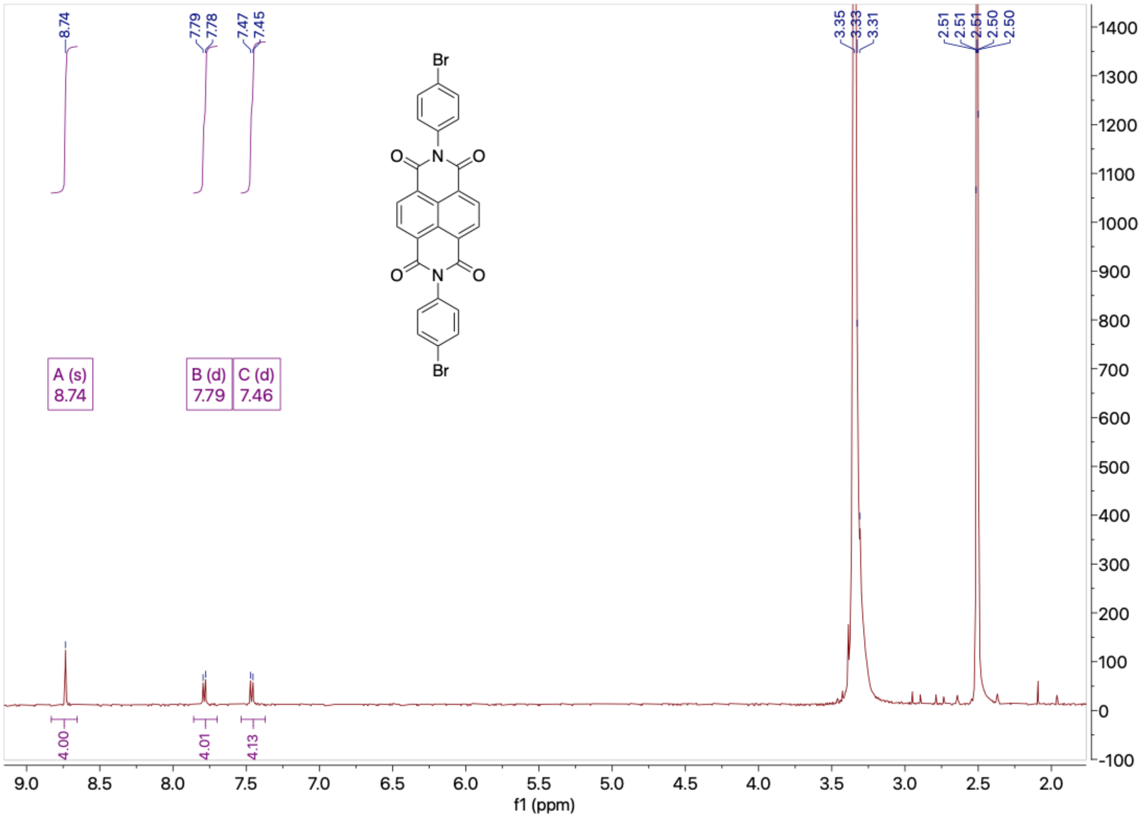

Supplementary Figure 14: ^{1}H NMR spectrum of 2,7-bis(4-bromphenyl)benzo[lmn][3,8]phenanthroline-1,3,6,8(2H,7H)-tetraone

NDI-(PhBr)-(Hex) precursor: 7-hexyl-1 *H*-isochromeno[6,5,4-*def*]isoquinoline-1,3,6,8(7 *H*)-tetraone:

A slightly modified synthetic protocol[2]: To a 1000 mL round bottom flask equipped with a magnetic stir bar napthalenetetracarboxylic dianhydride (10 g, 37.3 mmol, 1 eq.) suspended in distilled water and 170 mL of a 1 M KOH solution was added. The solution was left to stir at 60 °C for 30 minutes until all starting materials were fully dissolved. Afterwards, the pH of the solution was adjusted to around 5-6 using a solution of 1 M phosphoric acid. Then hexylamine (4.90 mL, 37.3 mmol, 1 eq.) was added in one portion and the pH of the solution was again adjusted to 5-6 using a 1 M solution of phosphoric acid. The reaction was then heated to reflux for 24 hours and then cooled to room temperature. The solution was filtered and then the filtrate was acidified using about 10-20 mL of glacial acetic acid. A white precipitate immediately formed, and the solution was filtered. The precipitate was washed excessively with distilled water. Afterwards, the filtered precipitate was dried in a high vacuum oven overnight where a white powder was received and used without any further purifications (11.4 g, 88% yield). ^{1}H NMR (500 MHz, DMSO) δ 8.52 (d, J = 7.5 Hz, 2H), 8.07 (d, J = 7.6 Hz, 2H), 4.04 (dd, J = 8.4, 6.7 Hz, 2H), 1.65 (ddd, J = 9.1, 6.2, 2.0 Hz, 2H), 1.37 – 1.26 (m, 6H), 0.94 – 0.81 (m, 3H). ^{13}C NMR (126 MHz, $CDCl_3$) δ 170.05, 163.51, 130.60, 128.96, 128.46, 125.98, 123.80, 31.43, 27.84, 26.64, 22.44, 14.39.

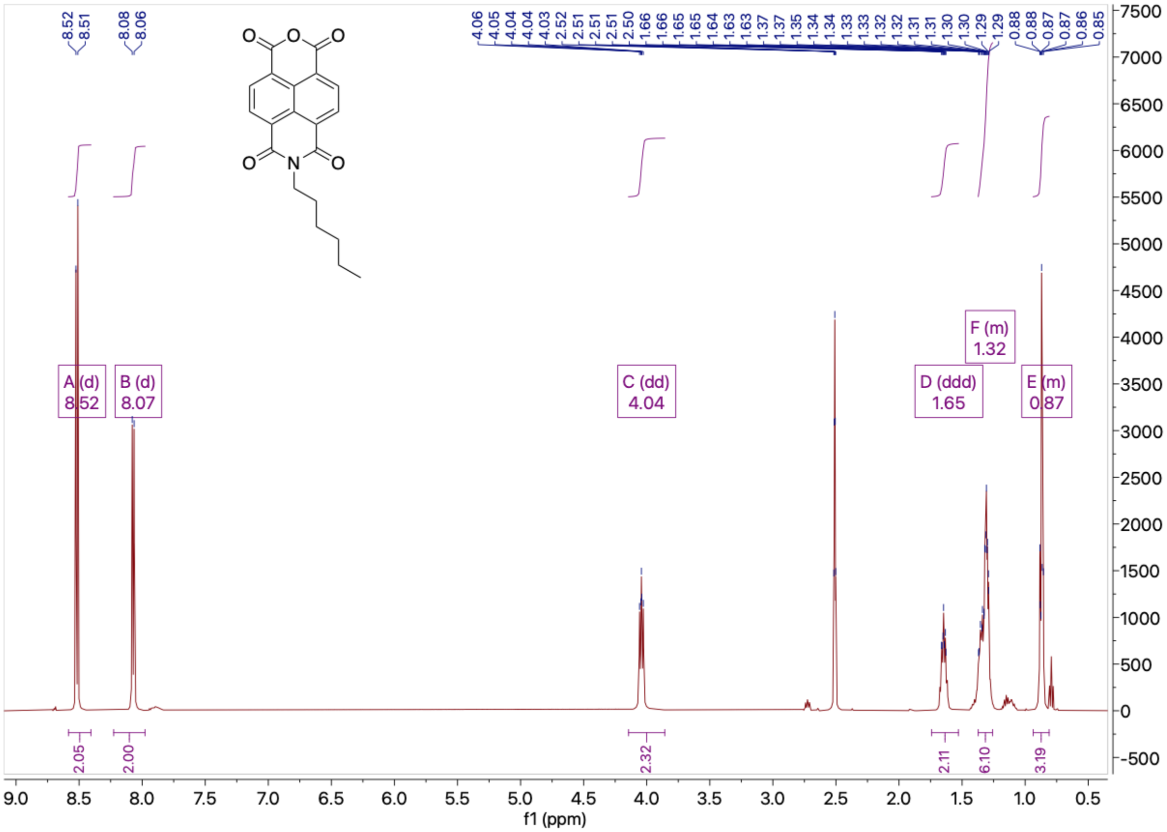

Supplementary Figure 15: ^{1}H NMR spectrum of 7-hexyl-1 *H*-isochromeno[6,5,4-*def]*isoquinoline-1,3,6,8(7 *H*)-tetraone.

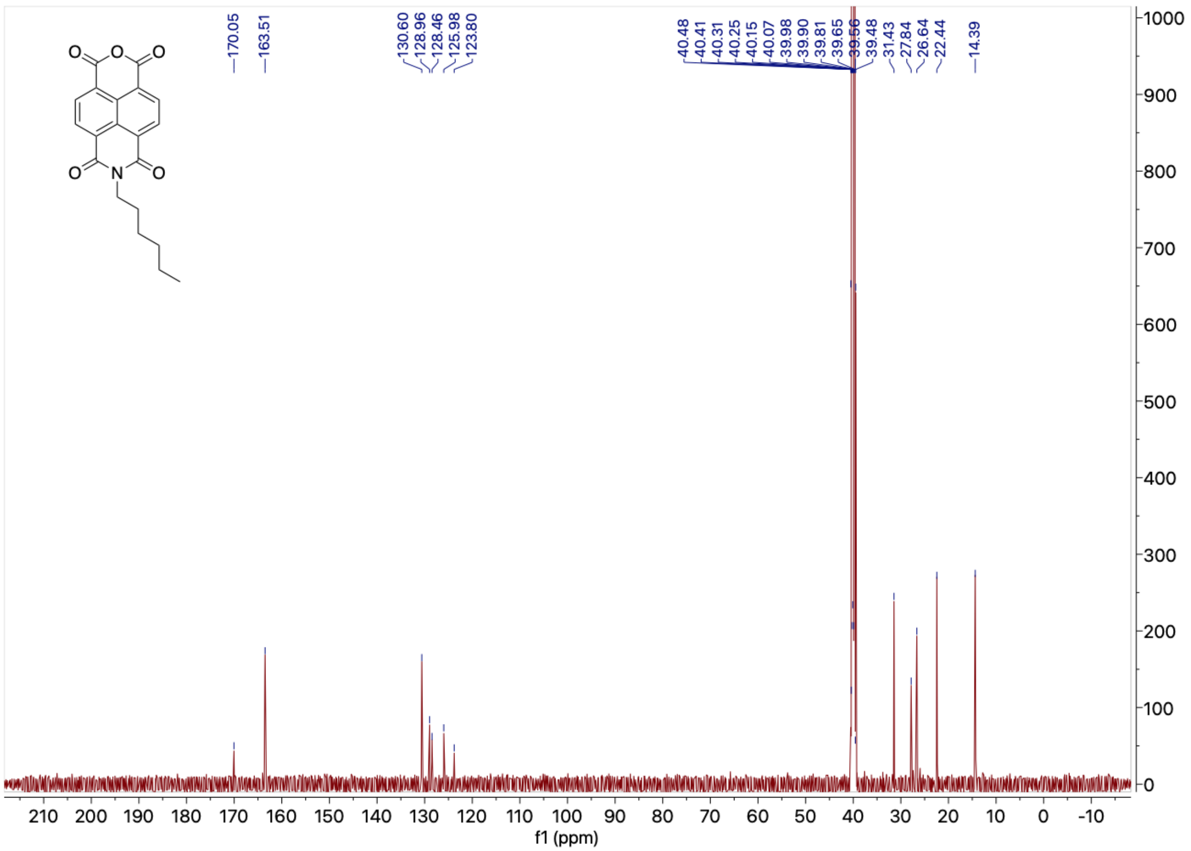

Supplementary Figure 16: ^{13}C NMR spectrum of 7-hexyl-1 *H*-isochromeno[6,5,4-*def]*isoquinoline-1,3,6,8(7 *H*)-tetraone.

NDI-(PhBr)-(Hex): 2-(4-bromophenyl)-7-hexylbenzo[*lmn*][3,8]phenanthroline-1,3,6,8(2*H*, 7*H*)-tetraone:

To a 250 mL round bottom flask charged with a magnetic stir bar 7-hexyl-1 *H*-isochromeno[6,5,4-*def]*isoquinoline-1,3,6,8(7 *H*)-tetraone (2.3g, 6.3 mmoles, 1 eq.) and 100 mL of glacial acetic acid were added. The solution was heated to 60 °C and stirred for about 10 minutes before 4-bromoaniline (2.3 g, 13.3 mmol, 1.5 eq) was added. The solution was further heated to 110 °C and left to stir overnight. Afterwards, the solution was then cooled to room temperature, precipitated into distilled water, and filtered. The precipitate was dried in under high vacuum and then purified by silica gel chromatography using a gradient of 100% DCM, 1:1 DCM: EtOAC mixture, and 100% EtOAC. A pure white powder was obtained (2.60 g, 82% yield). ^{1}H NMR (500 MHz, $CDCl_3$) δ 8.83 (s, 4H), 7.79 – 7.67 (m, 2H), 7.26 – 7.19 (m, 2H), 4.33 – 4.09 (m, 2H), 1.78 (t, J = 7.7 Hz, 2H), 1.47 (d, J = 7.8 Hz, 2H), 1.43 – 1.30 (m, 4H), 0.97 – 0.87 (m, 3H). ^{13}C NMR (126 MHz, $CDCl_3$) δ 162.85, 162.71, 133.55, 132.84, 131.151, 131.05, 130.25, 127.14, 127.07, 126.86, 126.45, 123.33, 41.11, 31.50, 28.04, 26.76, 22.56, 14.06.

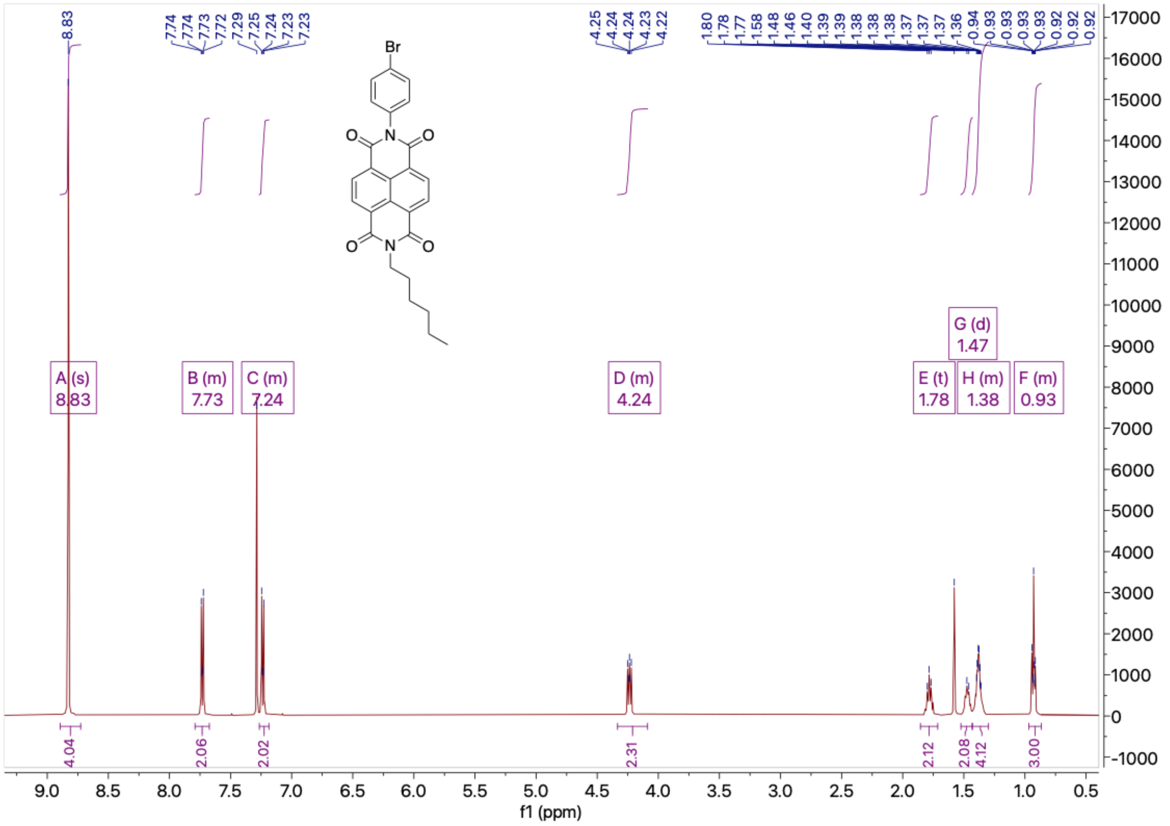

Supplementary Figure 17: ^{1}H NMR spectrum of 2-(4-bromophenyl)-7-hexylbenzo[*lmn*][3,8]phenanthroline-1,3,6,8(2*H*, 7*H*)-tetraone.

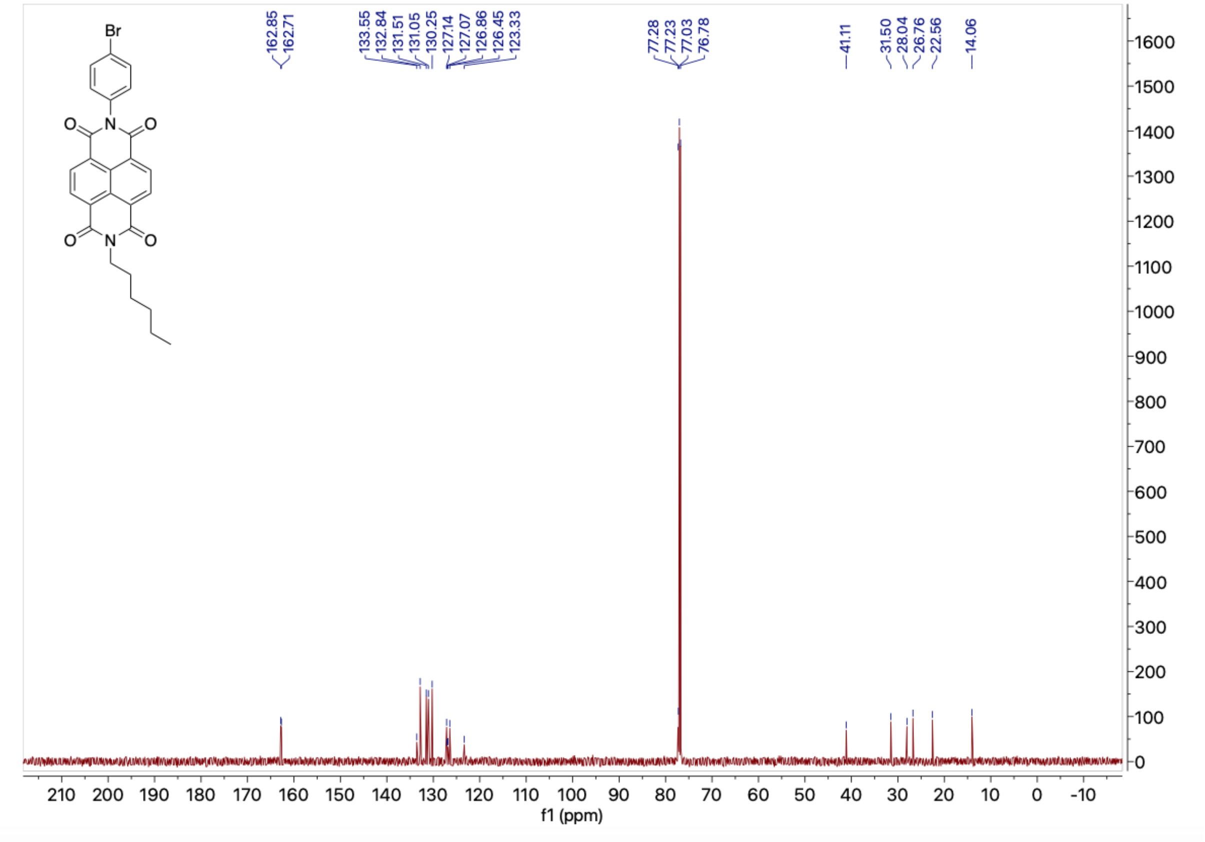

Supplementary Figure 18: ^{13}C NMR spectrum of 2-(4-bromophenyl)-7-hexylbenzo[*lmn*][3,8]phenanthroline-1,3,6,8(2*H*, 7*H*)-tetraone.

NDI-(BzPA)-(Hex) precursor: Diethyl (4-(7-hexyl-1,3,6,8-tetraoxo-3,6,7,8-tetrahydrobenzo[*lmn*][3,8]phenanthroline-2(1 *H*)-yl)benzyl)phosphonate:

To a 250 mL round bottom flask charged with a magnetic stir bar 7-hexyl-1 *H*-isochromeno[6,5,4-*def]*isoquinoline-1,3,6,8(7 *H*)-tetraone (2.0 g, 5.7 mmol, 1 eq.) and 100 mL of glacial acetic acid were added. The solution was heated to 60 °C and stirred for about 10 minutes before diethyl (4-aminobenzyl)phosphonate (2.8 g, 11.4 mmol, 1.5 eq) was added. The solution was further heated to 110°C and left to stir overnight. Afterwards, the solution was then cooled to room temperature, precipitated into distilled water, and filtered. The precipitate was dried in under high vacuum and then purified by silica gel chromatography using a gradient of 100% DCM, 1:1 DCM: EtOAC mixture, and 100% EtOAC. A pure bright yellow powder was obtained (2.10 g, 64% yield). ^{1}H NMR (500 MHz, $CDCl_3$) δ 8.82 (s, 4H), 7.54 (dd, *J* = 8.5, 2.5 Hz, 2H), 7.32 – 7.29 (m, 2H), 4.28 – 4.20 (m, 2H), 4.10 (ddd, *J* = 8.5, 7.0, 1.4 Hz, 4H), 3.37 – 3.07 (m, 2H), 1.78 (d, *J* = 7.7 Hz, 2H), 1.53 – 1.44 (m, 2H), 1.38 (dt, *J* = 6.5, 3.2 Hz, 4H), 1.31 (t, *J* = 7.1 Hz, 6H), 0.93 (t, *J* = 7.0 Hz, 3H). ^{31}P NMR (202 MHz, $CDCl_3$) δ 26.23. ^{13}C NMR (126 MHz, $CDCl_3$) δ 162.97, 162.77, 133.37, 132.95, 131.35, 131.03, 130.95, 130.90, 128.65, 128.62, 127.09, 127.01, 126.87, 126.70, 62.36, 62.31, 41.08, 34.23, 33.13, 351.50, 28.04, 26.75, 22.55, 16.45, 16.40, 14.04.

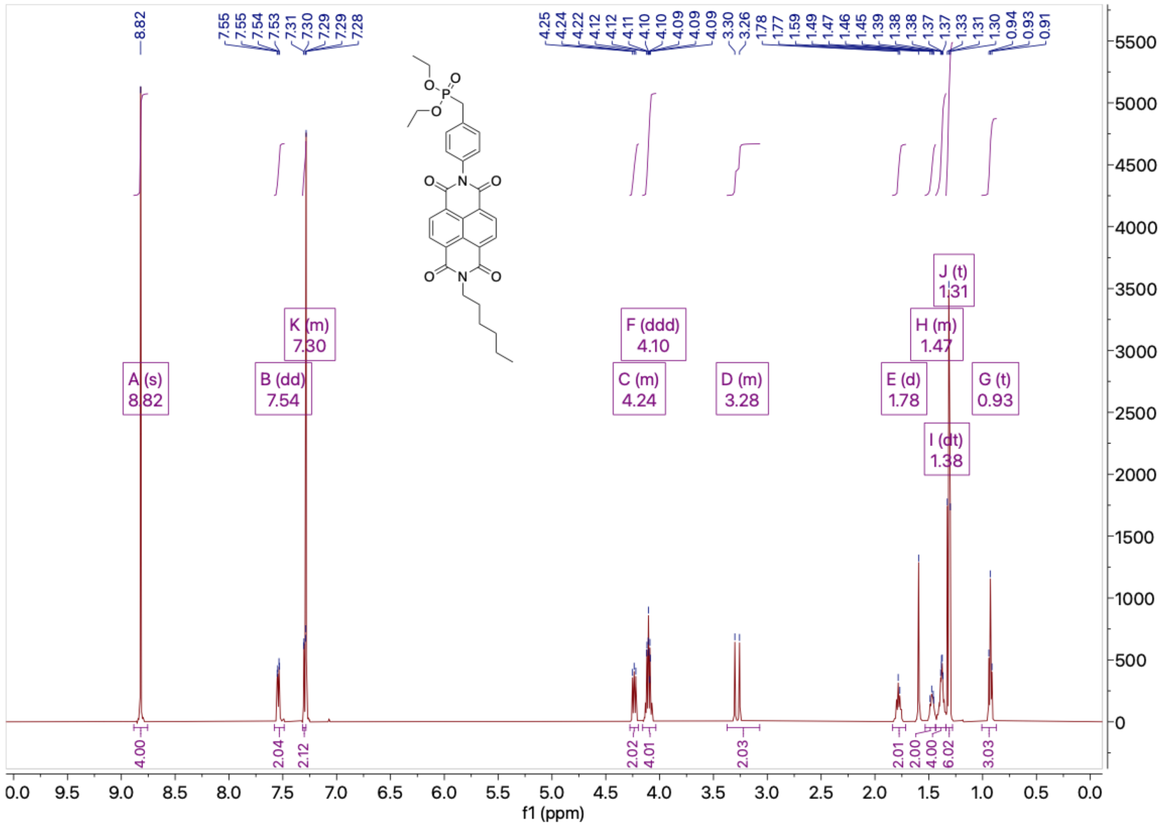

Supplementary Figure 19: ^{1}H NMR spectrum of diethyl (4-(7-hexyl-1,3,6,8-tetraoxo-3,6,7,8-tetrahydrobenzo[*lmn*][3,8]phenanthroline-2(1 *H*)-yl)benzyl)phosphonate.

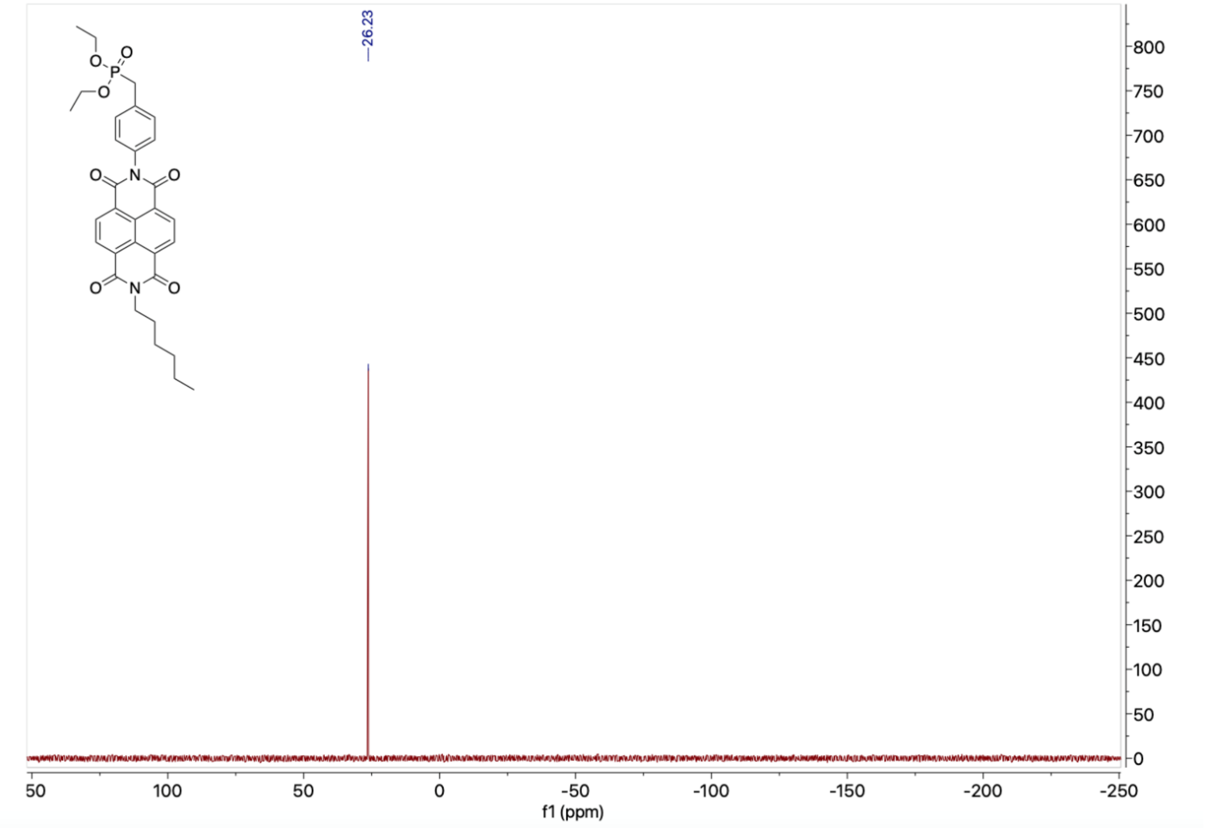

Supplementary Figure 20: ^{31}P NMR spectrum of diethyl (4-(7-hexyl-1,3,6,8-tetraoxo-3,6,7,8-tetrahydrobenzo[*lmn*][3,8]phenanthroline-2(1 *H*)-yl)benzyl)phosphonate

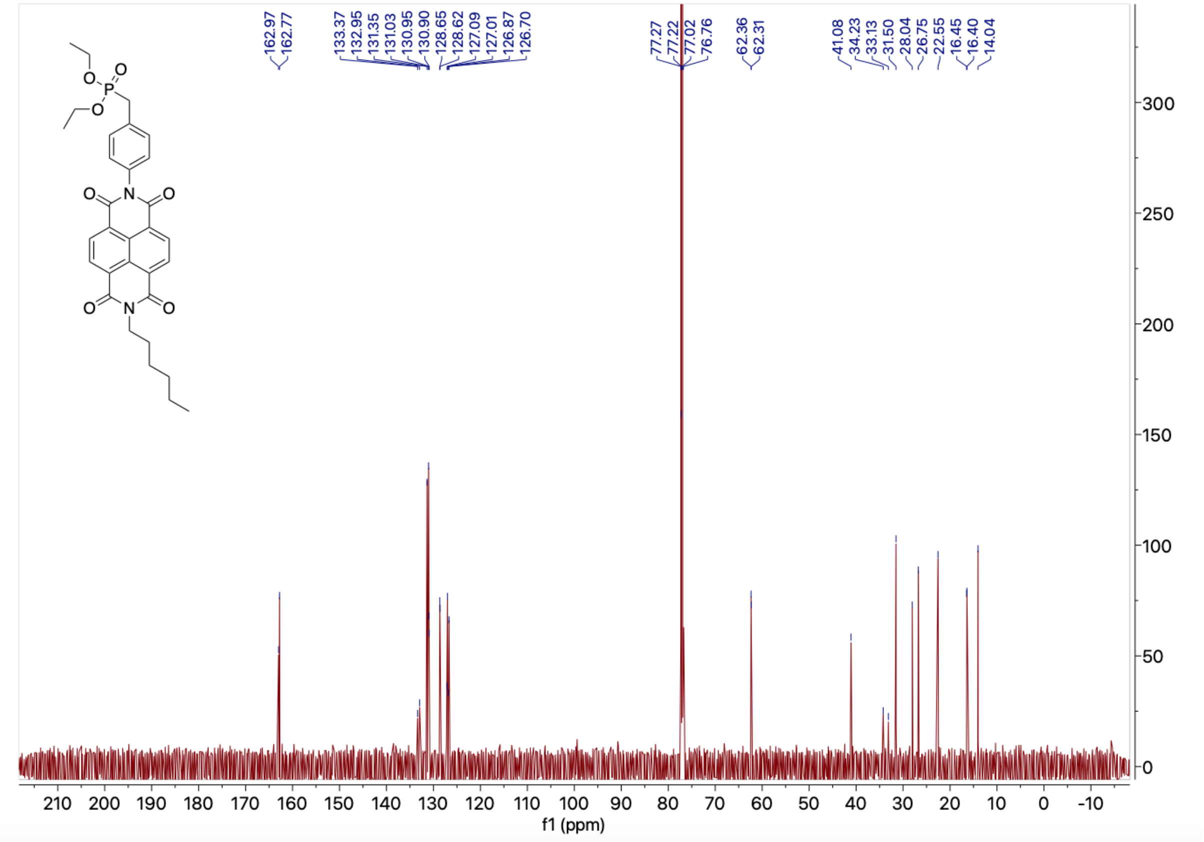

Supplementary Figure 21: ^{13}C NMR spectrum of diethyl (4-(7-hexyl-1,3,6,8-tetraoxo-3,6,7,8-tetrahydrobenzo[*lmn*][3,8]phenanthroline-2(1 *H*)-yl)benzyl)phosphonate.

NDI-(BzPA)-(Hex) : (4-(7-hexyl-1,3,6,8-tetraoxo-3,6,7,8-tetrahydrobenzo[*lmn*][3,8]phenanthroline-2(1 *H*)-yl)benzyl)phosphonic acid:

To a 100 mL round bottom flask charged with a magnetic stir bar diethyl (4-(7-hexyl-1,3,6,8-tetraoxo-3,6,7,8-tetrahydrobenzo[*lmn*][3,8]phenanthroline-2(1 *H*)-yl)benzyl)phosphonate (2.0 g, 3.4 mmol, 1 eq.) and 25 mL of anhydrous dichloromethane were added under an inert atmosphere. After stirring for 10 minutes at room temperature, bromotrimethylsilane (5.2 g, 34.1 mmol, 10 eq.) was added and the reaction was left to stir overnight at room temperature. After 12 hours, 3 mL of methanol was added and left to stir for an additional 3 hours. The solution was then concentrated to complete dryness and the powder was suspended in methanol and filtered. The product was then dried under high vacuum to receive a white powder (1.77 g, 100% yield). ^{1}H NMR (500 MHz, DMSO) δ 8.82 – 8.49 (m, 4H), 7.42 (dd, J = 8.3, 2.4 Hz, 2H), 7.34 (d, J = 8.2 Hz, 2H), 4.09 – 4.05 (m, 2H), 3.12 – 3.03 (m, 2H), 1.68 (dd, J = 8.8, 6.4 Hz, 2H), 1.43 – 1.36 (m, 2H), 1.33 (tt, J = 5.9, 2.9 Hz, 4H), 0.88 (s, 3H). ^{31}P NMR (202 MHz, DMSO) δ 21.25. ^{13}C NMR (126 MHz, DMSO) δ 163.41, 1631.11, 135.18, 135.11, 133.90, 130.92, 130.90, 130.78, 130.73, 128.96, 127.35, 127.02, 126.82, 126.69, 36.20, 35.15, 31.42, 27.81, 26.65, 22.44, 14.38.

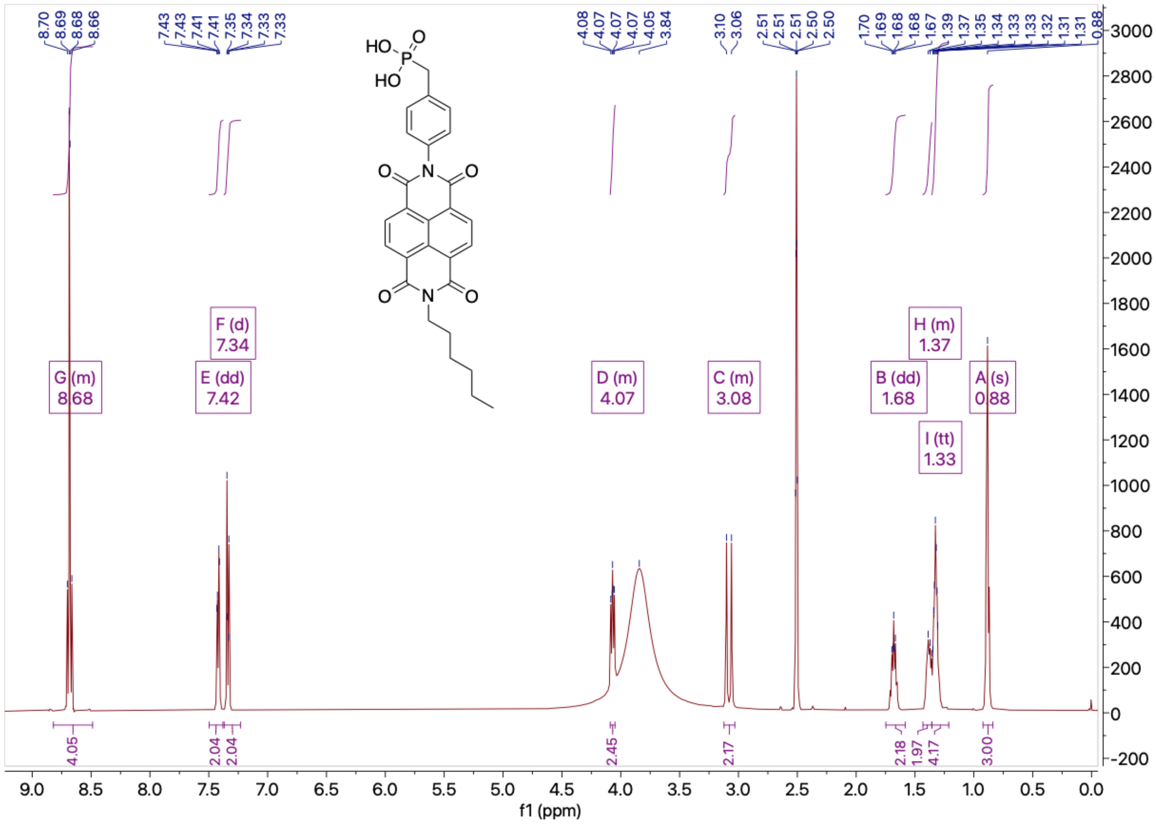

Supplementary Figure 22: ^{1}H NMR spectrum of (4-(7-hexyl-1,3,6,8-tetraoxo-3,6,7,8-tetrahydrobenzo[*lmn*][3,8]phenanthroline-2(1 *H*)-yl)benzyl)phosphonic acid

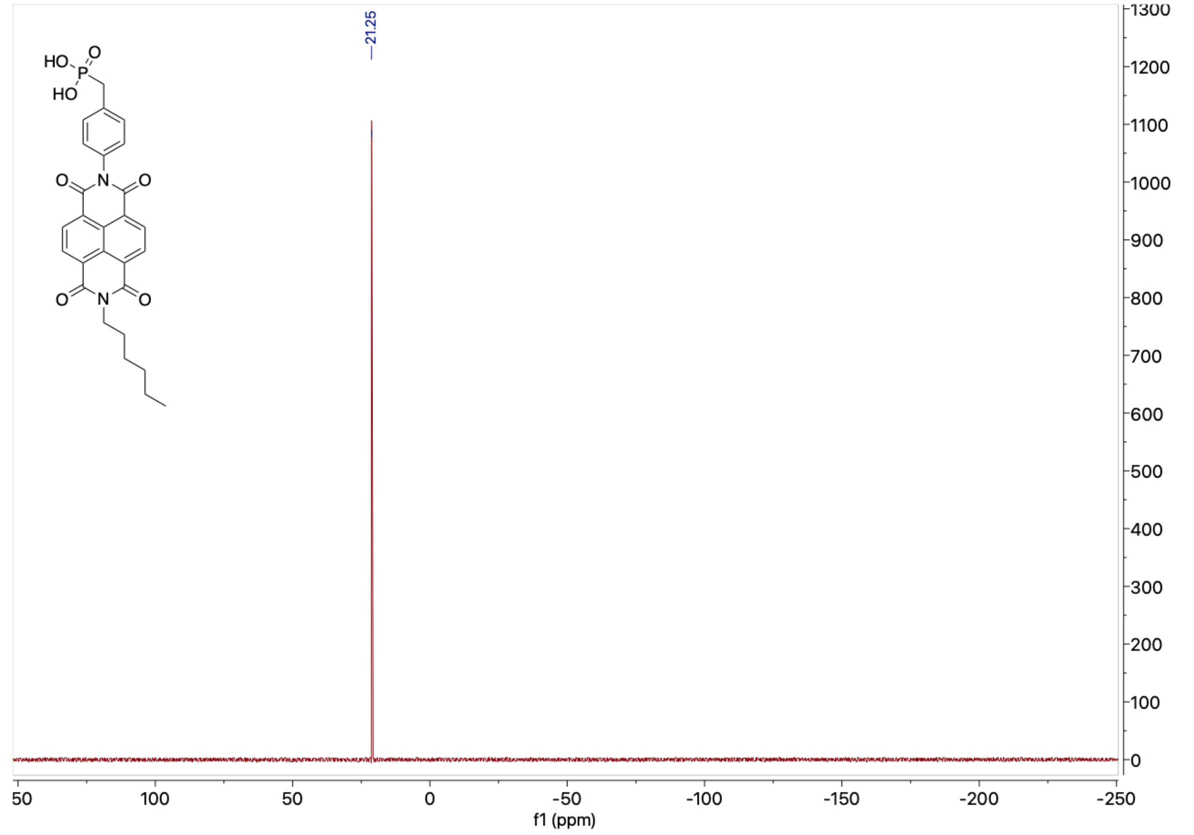

Supplementary Figure 23: ^{31}P NMR spectrum of (4-(7-hexyl-1,3,6,8-tetraoxo-3,6,7,8-tetrahydrobenzo[*lmn*][3,8]phenanthroline-2(1 *H*)-yl)benzyl)phosphonic acid.

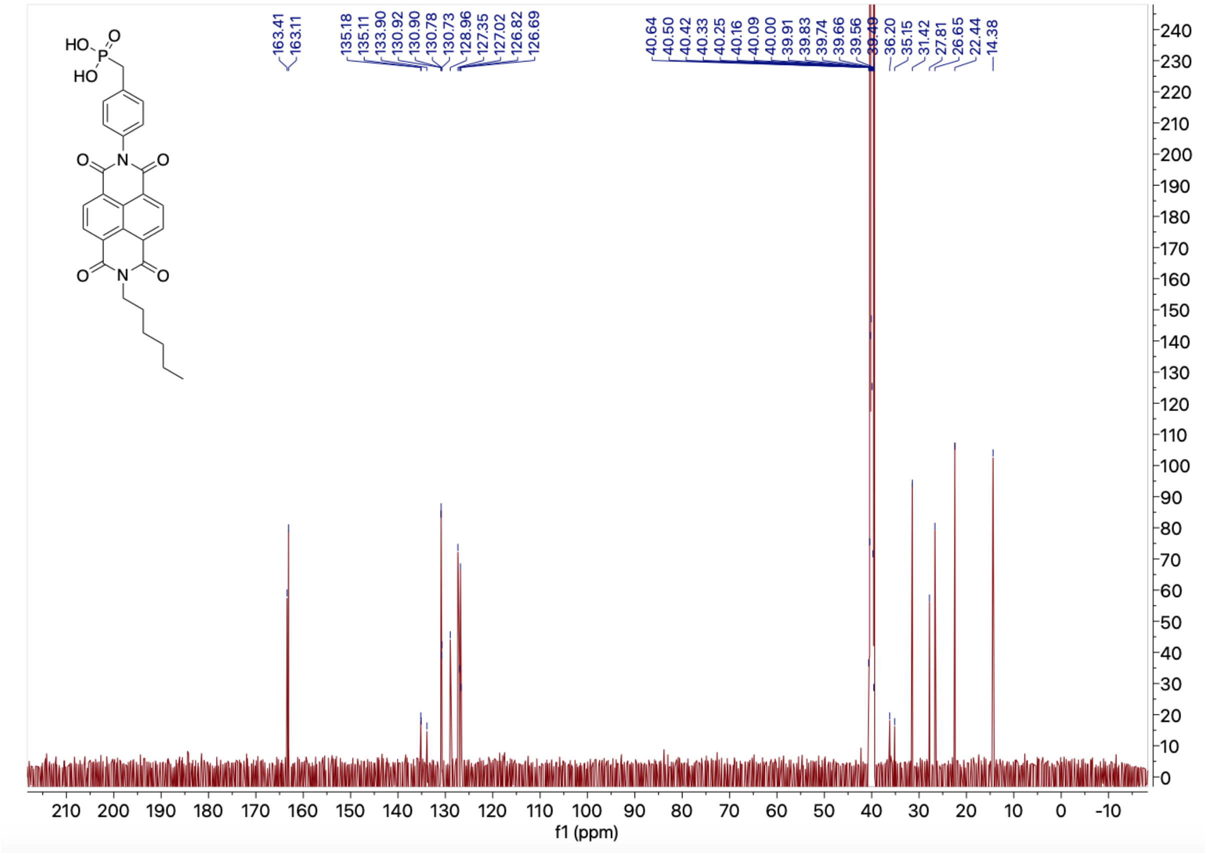

Supplementary Figure 24: ^{13}C NMR spectrum of (4-(7-hexyl-1,3,6,8-tetraoxo-3,6,7,8-tetrahydrobenzo[*lmn*][3,8]phenanthroline-2(1 *H*)-yl)benzyl)phosphonic acid.

NDI-(BzPA)-(PhBr) precursor: 7-(4-bromophenyl)-1H-isochromeno[6,5,4-def]isoquinoline-1,3,6,8(7 H)-tetraone:

A slightly modified synthetic protocol[2]: To a 1000 mL round bottom flask equipped with a magnetic stir bar napthalenetetracarboxylic dianhydride (10 g, 37.3 mmol, 1 eq.) suspended in distilled water and 170 mL of a 1 M KOH solution was added. The solution was left to stir at 60 °C for 30 minutes until all starting materials were fully dissolved. Afterwards, the pH of the solution was adjusted to around 5-6 using a solution of 1 M phosphoric acid. Then 4-bromoaniline (6.4 g, 37.3 mmol, 1 eq.) was added in one portion and the pH of the solution was again adjusted to 5-6 using a 1 M solution of phosphoric acid. The reaction was then heated to reflux for 24 hours and then cooled to room temperature. The solution was filtered and then the filtrate was acidified using about 10-20 mL of glacial acetic acid. A white precipitate immediately formed, and the solution was filtered. The precipitate was washed excessively with distilled water. Afterwards, the filtered precipitate was dried in a high vacuum oven overnight where a white powder was received and used without any further purifications (8.7 g, 55% yield).

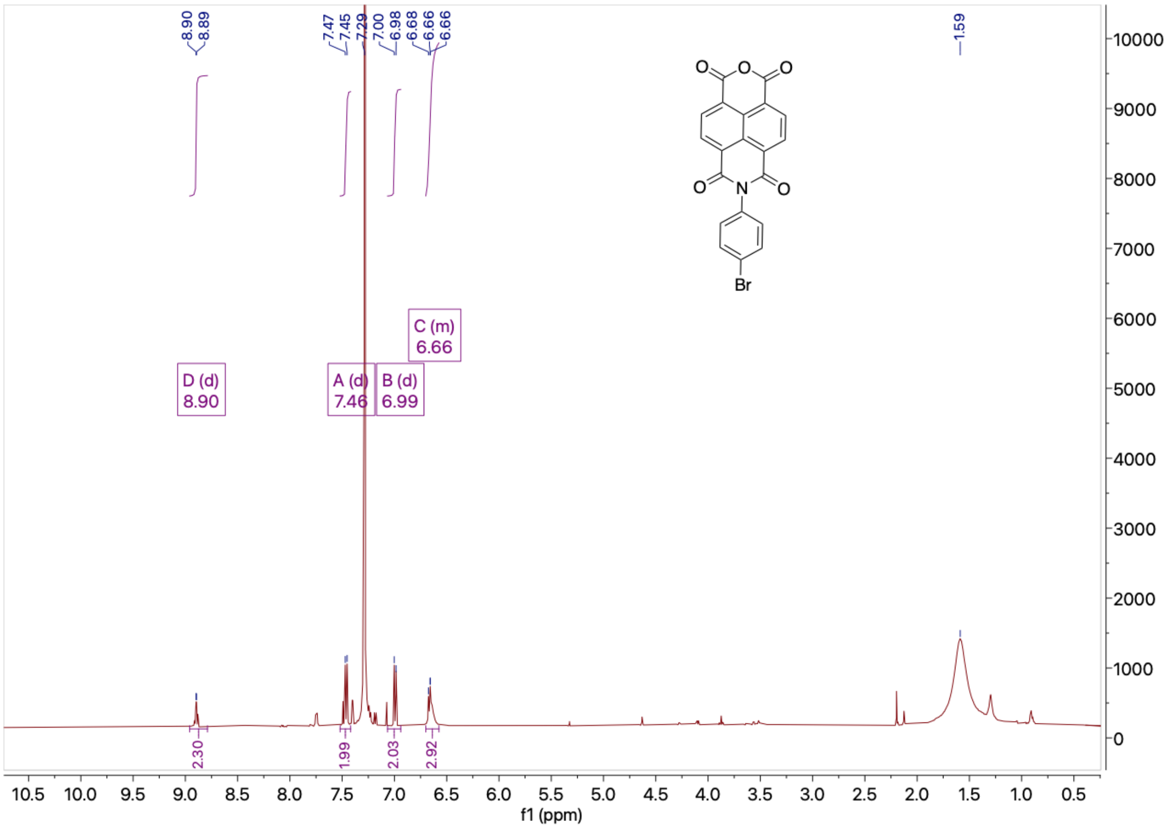

Supplementary Figure 25: ^{1}H NMR spectrum of 7-(4-bromophenyl)-1H-isochromeno[6,5,4-def]isoquinoline-1,3,6,8(7 H)-tetraone.

NDI-(BzPA)-(PhBr) precursor: Diethyl (4-(7-(4-bromophenyl)-1,3,6,8-tetraoxo-3,6,7,8-tetrahydrobenzo[*lmn*][3,8]phenanthroline-2(1 *H*)-yl)benzyl)phosphonate:

To a 250 mL round bottom flask charged with a magnetic stir bar 7-(4-bromophenyl)-1H-isochromeno[6,5,4-def]isoquinoline-1,3,6,8(7 H)-tetraone (2.0g, 4.7 mmol, 1 eq.) and 100 mL of glacial acetic acid were added. The solution was heated to 60 °C and stirred for about 10 minutes before diethyl (4-aminobenzyl)phosphonate (2.3 g, 9.47 mmoles, 1.5 eq) was added. The solution was further heated to 110 °C and left to stir overnight. Afterwards, the solution was then cooled to room temperature, precipitated into distilled water, and filtered. The precipitate was dried in under high vacuum and then purified by silica gel chromatography using a gradient of 100% DCM, 1:1 DCM: EtOAC mixture, and 1:3 MeOH:DCM. A yellow powder was obtained (1.5 g, 50% yield). ^{1}H NMR (500 MHz, $CDCl_3$) δ 8.87 (s, 4H), 7.74 (d, J = 8.2 Hz, 2H), 7.56 (dd, J = 8.3, 2.3 Hz, 2H), 7.32 (d, J = 7.9 Hz, 2H), 7.26 (d, J = 8.2 Hz, 2H), 4.12 (q, J = 7.3 Hz, 4H), 3.40 – 3.15 (m, 2H), 1.32 (t, J = 7.0 Hz, 6H). ^{31}P NMR (202 MHz, $CDCl_3$) δ 26.22. ^{13}C NMR (126 MHz, $CDCl_3$) δ 162.83, 162.76, 132.86, 131.60, 131.46, 131.00, 130.24, 128.62, 127.18, 126.81, 62.38, 30.93, 16.45, 16.40.

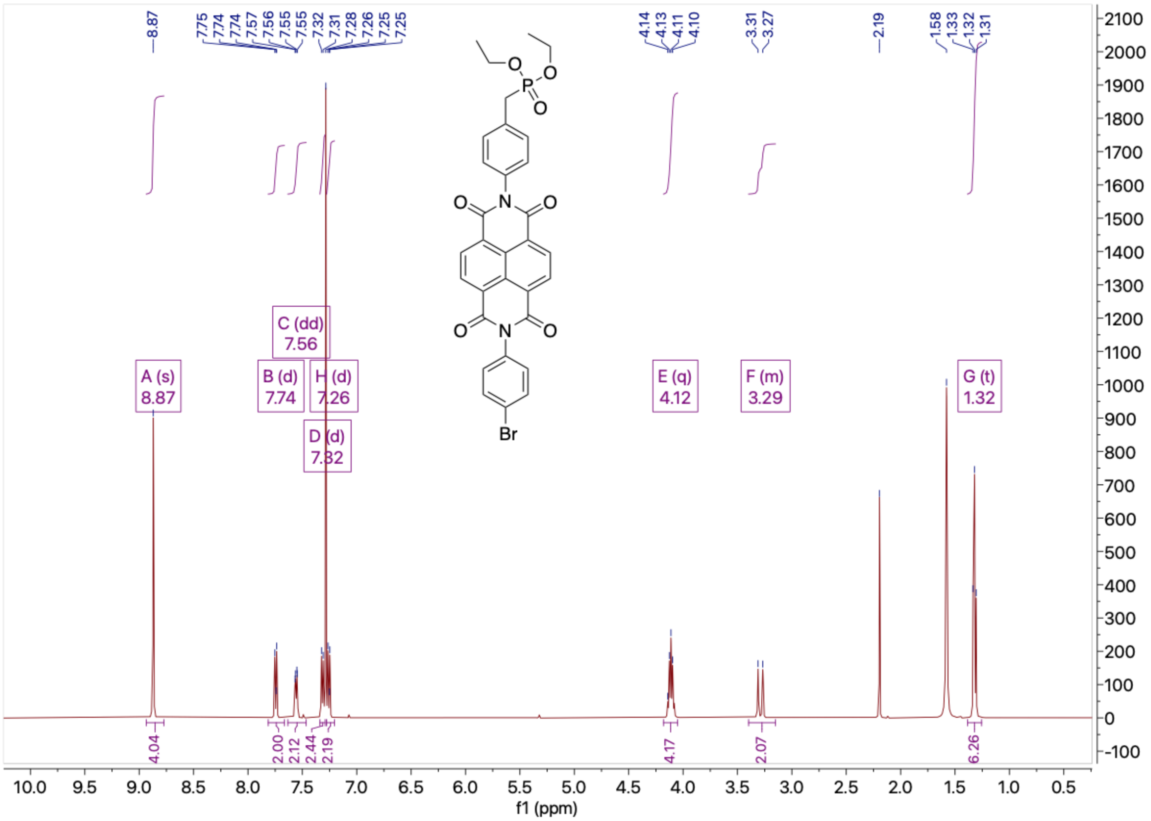

Supplementary Figure 26: ^{1}H NMR spectrum of diethyl (4-(7-(4-bromophenyl)-1,3,6,8-tetraoxo-3,6,7,8-tetrahydrobenzo[*lmn*][3,8]phenanthroline-2(1 *H*)-yl)benzyl)phosphonate.

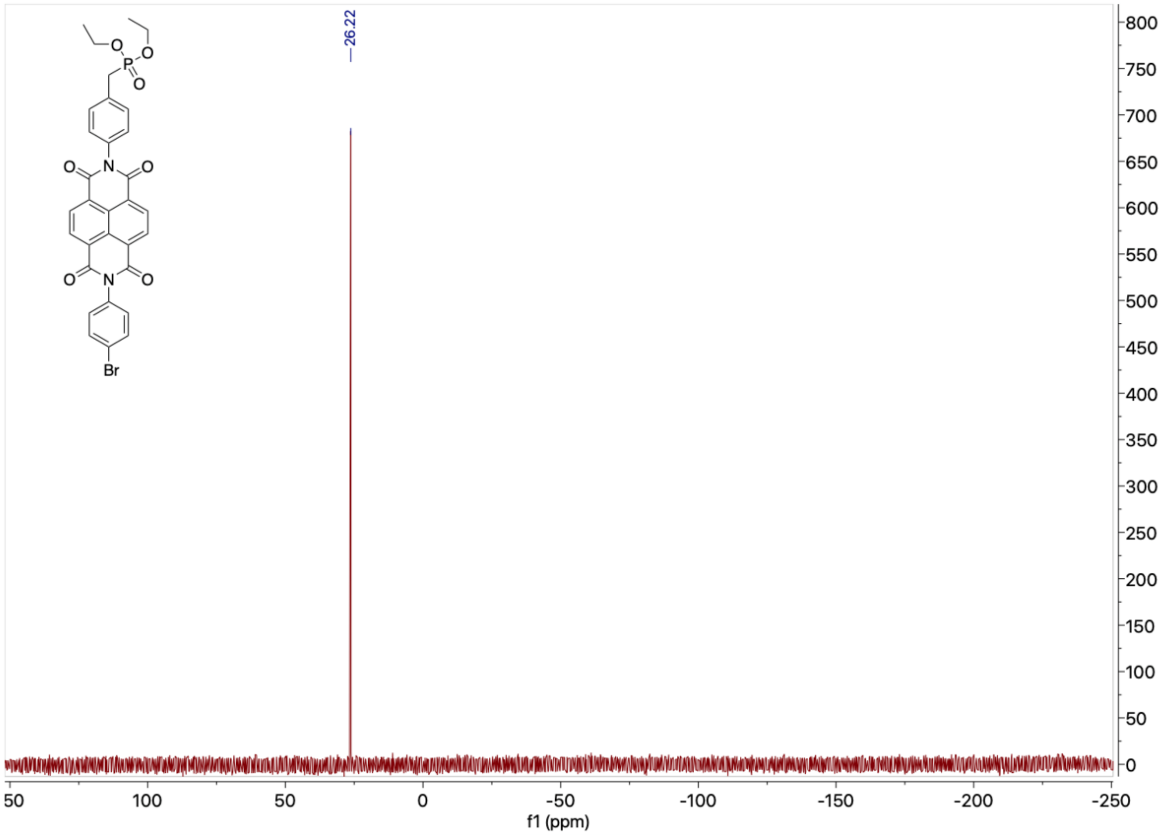

Supplementary Figure 27: ^{31}P NMR spectrum of diethyl (4-(7-(4-bromophenyl)-1,3,6,8-tetraoxo-3,6,7,8-tetrahydrobenzo[*lmn*][3,8]phenanthroline-2(1 *H*)-yl)benzyl)phosphonate.

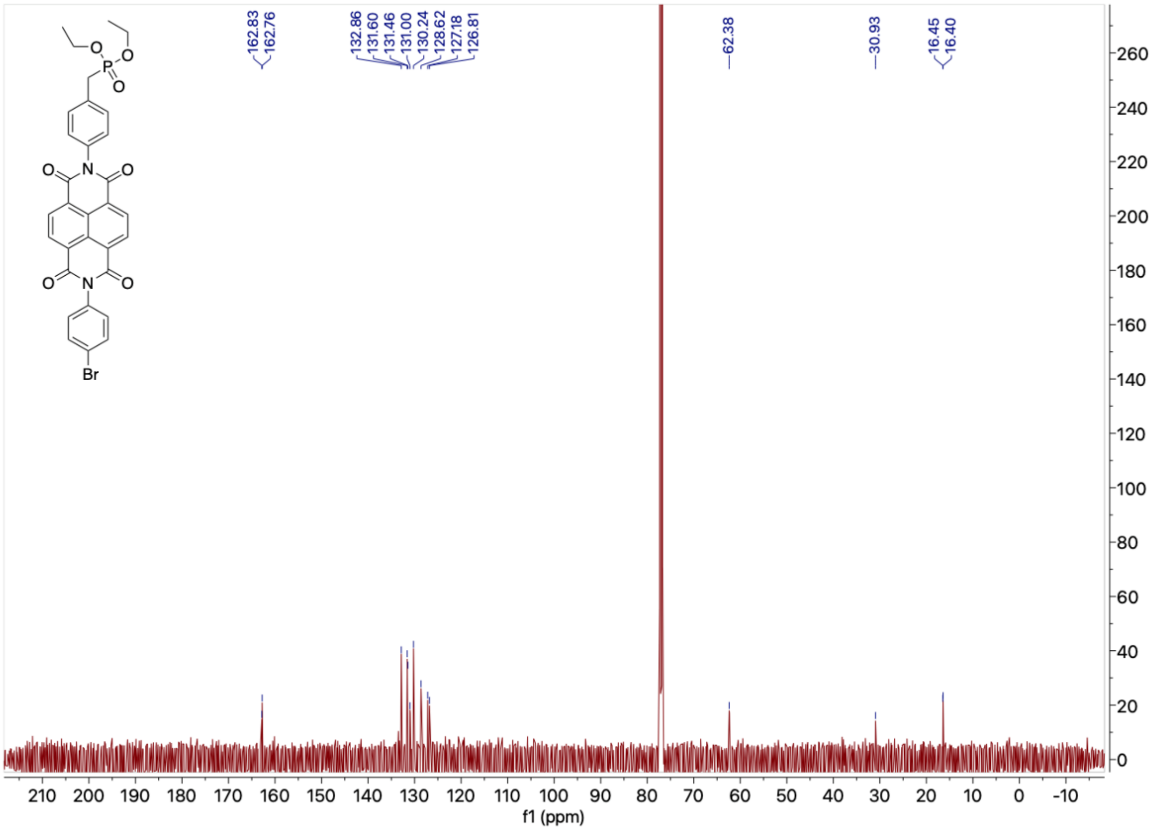

Supplementary Figure 28: ^{13}C NMR spectrum of diethyl (4-(7-(4-bromophenyl)-1,3,6,8-tetraoxo-3,6,7,8-tetrahydrobenzo[*lmn*][3,8]phenanthroline-2(1 *H*)-yl)benzyl)phosphonate.

NDI-(BzPA)-(PhBr): Diethyl (4-(7-(4-bromophenyl)-1,3,6,8-tetraoxo-3,6,7,8-tetrahydrobenzo[*lmn*][3,8]phenanthroline-2(1 *H*)-yl)benzyl)phosphonic acid:

To a 100 mL round bottom flask charged with a magnetic stir bar diethyl (4-(7-(4-bromophenyl)-1,3,6,8-tetraoxo-3,6,7,8-tetrahydrobenzo[*lmn*][3,8]phenanthroline-2(1 *H*)-yl)benzyl)phosphonate (1.4 g, 2.2 mmol, 1 eq.) and 25 mL of anhydrous dichloromethane were added under an inert atmosphere. After stirring for 10 minutes at room temperature, bromotrimethylsilane (3.3 g, 21.6 mmol, 10 eq.) was added and the reaction was left to stir overnight at room temperature. After 12 hours, 3 mL of methanol was added and left to stir for an additional 3 hours. The solution was then concentrated to complete dryness and the powder was suspended in methanol and filtered. The product was then dried under high vacuum to receive a yellow powder (1.10g, 100% yield). ^{1}H NMR (500 MHz, DMSO) δ 8.73 (s, 4H), 7.78 (d, J = 8.4 Hz, 2H), 7.48 – 7.40 (m, 4H), 7.36 (d, J = 8.0 Hz, 2H), 3.06 (s, 2H). ^{31}P NMR (202 MHz, DMSO) δ 21.22. ^{13}C NMR (126 MHz, DMSO) δ 163.47, 163.34, 135.48, 133.92, 132.51, 131.83, 130.94, 130.79, 130.74, 128.99, 127.55, 127.38, 127.17, 122.21.

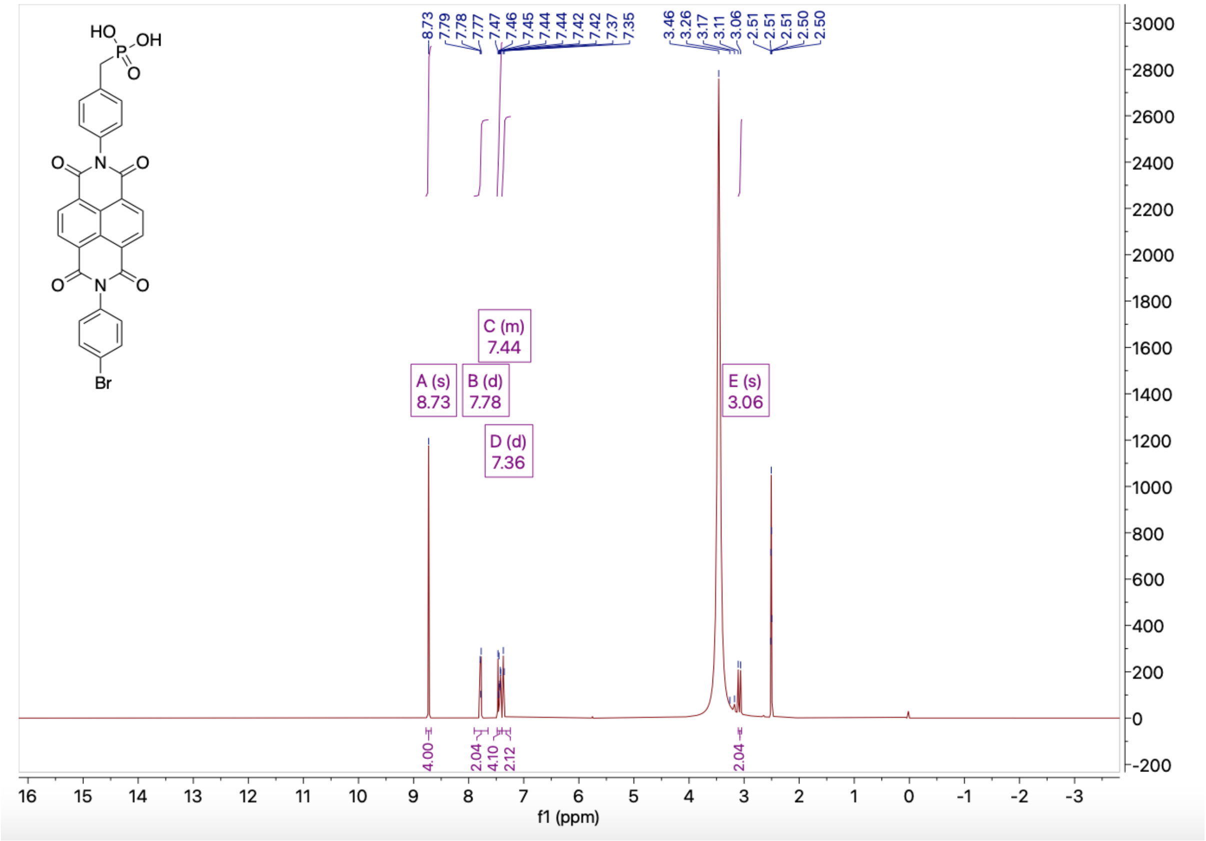

Supplementary Figure 29: ^{1}H NMR spectrum of diethyl (4-(7-(4-bromophenyl)-1,3,6,8-tetraoxo-3,6,7,8-tetrahydrobenzo[*lmn*][3,8]phenanthroline-2(1 *H*)-yl)benzyl)phosphonic acid.

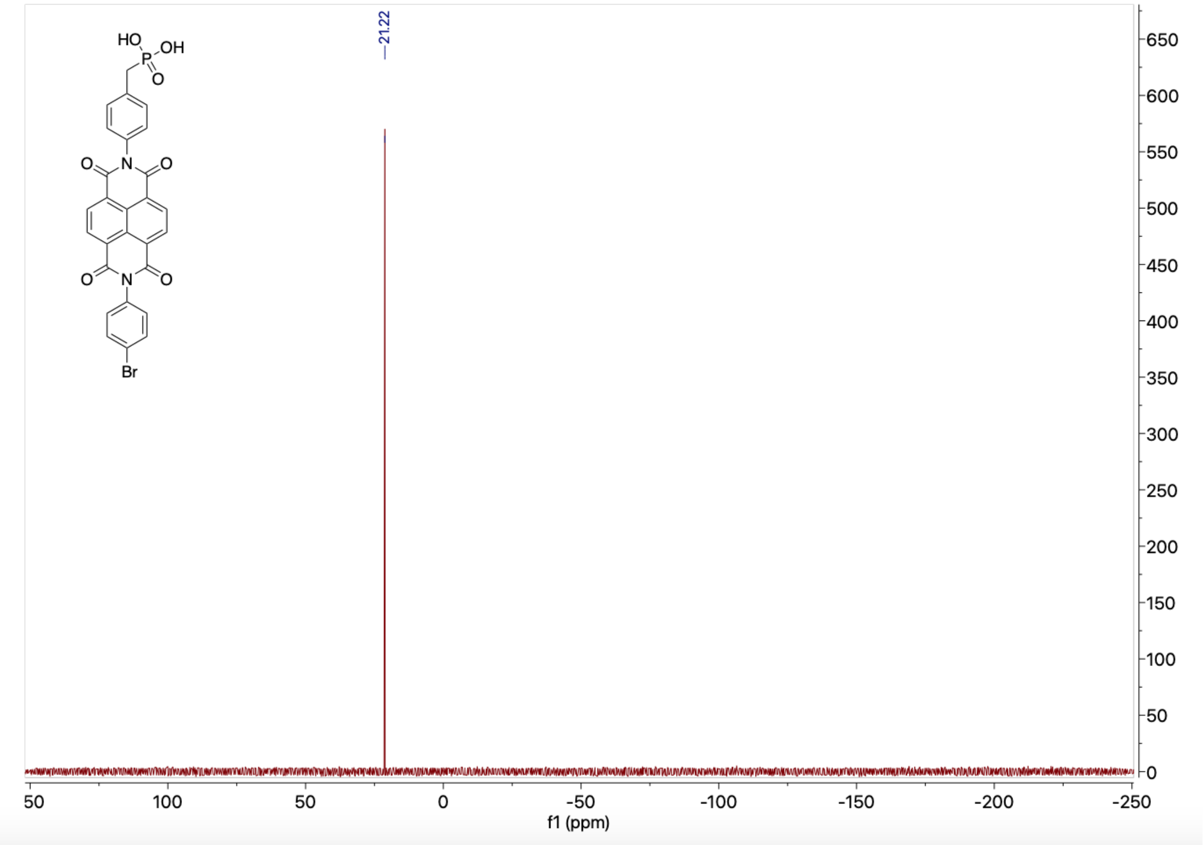

Supplementary Figure 30: ^{31}P NMR spectrum of diethyl (4-(7-(4-bromophenyl)-1,3,6,8-tetraoxo-3,6,7,8-tetrahydrobenzo[*lmn*][3,8]phenanthroline-2(1 *H*)-yl)benzyl)phosphonic acid.

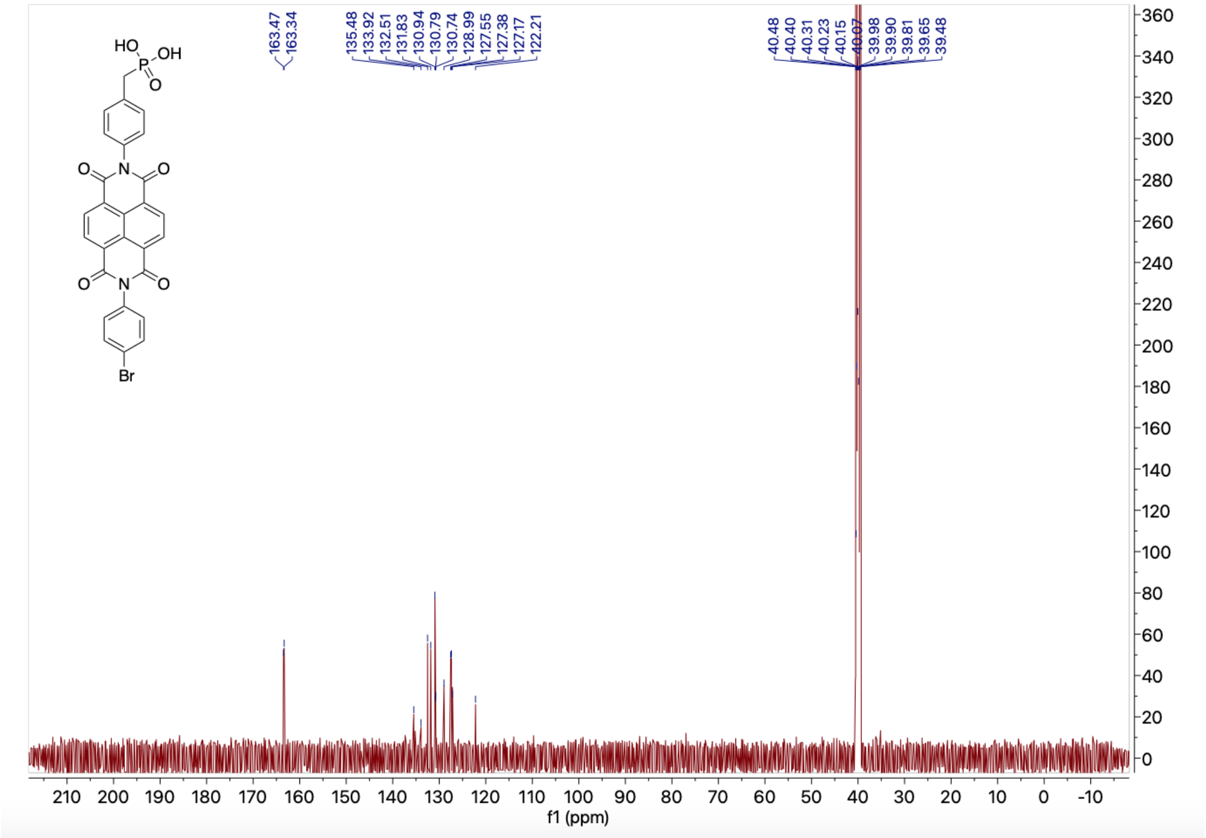

Supplementary Figure 31: ^{13}C NMR spectrum of diethyl (4-(7-(4-bromophenyl)-1,3,6,8-tetraoxo-3,6,7,8-tetrahydrobenzo[*lmn*][3,8]phenanthroline-2(1 *H*)-yl)benzyl)phosphonic acid.

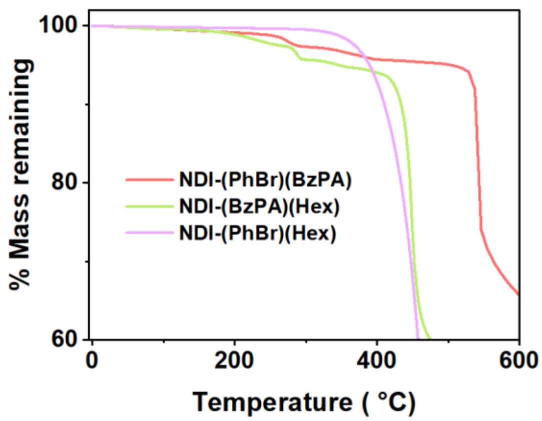

Supplementary Figure 32: Thermograms for NDI-(PhBr)(BzPA), NDI-(BzPA)(Hex) and NDI-(PhBr)(Hex).

‘

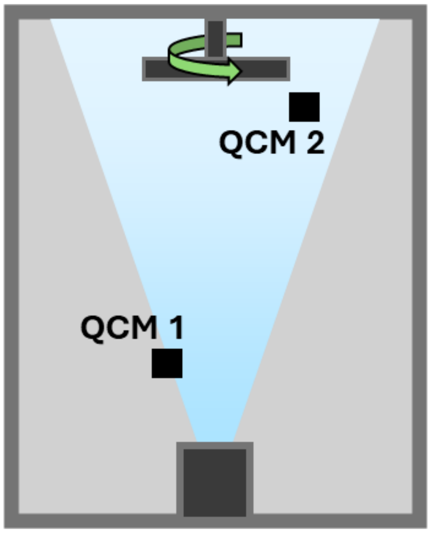

Supplementary Figure 33: Schematic of the evaporation chamber used throughout this work.

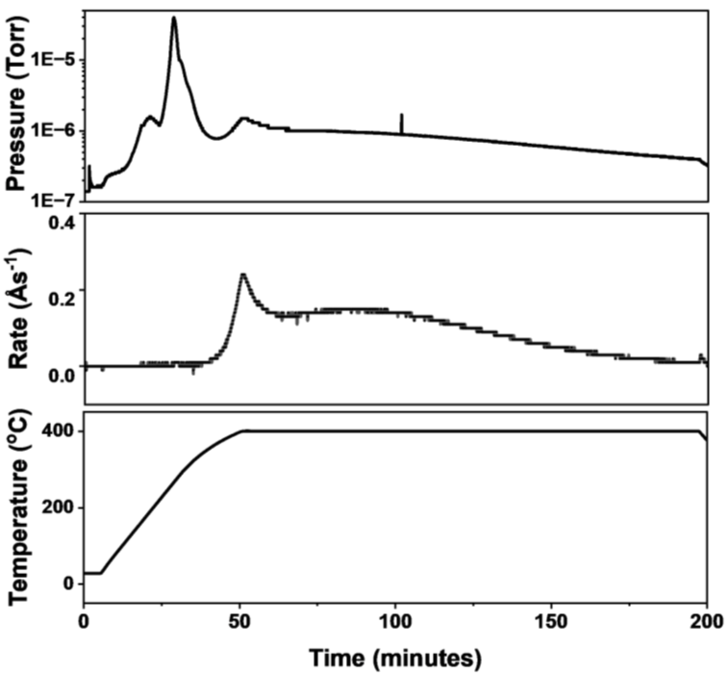

Supplementary Figure 34: Evaporation data recorded during thermal evaporation of NDI-(EtPA)$_2$ **(Top):** Pressure, **(Middle):** Deposition rate, **(Bottom):** Temperature.

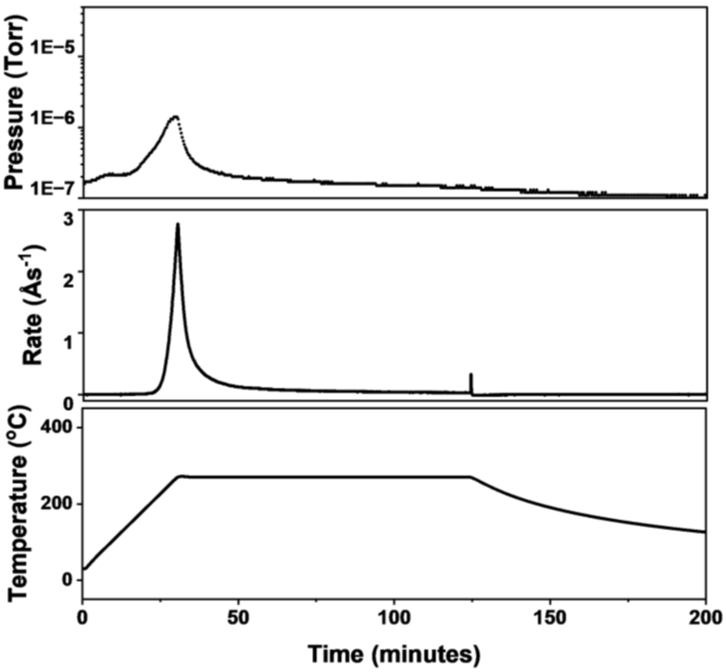

Supplementary Figure 35: Evaporation data recorded during thermal evaporation of NDI-(PhBr)$_2$ **(Top):** Pressure, **(Middle):** Deposition rate, **(Bottom):** Temperature.

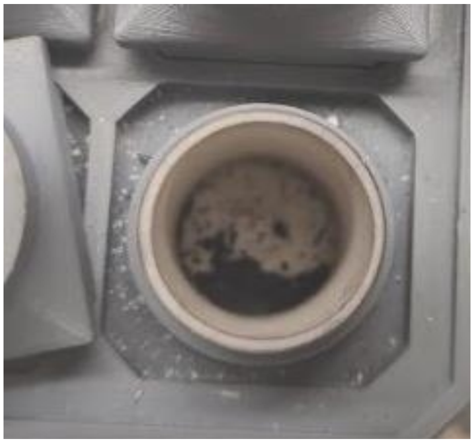

Supplementary Figure 36: Residual powder remaining in crucible after the deposition of NDI-(EtPA)$_2$.

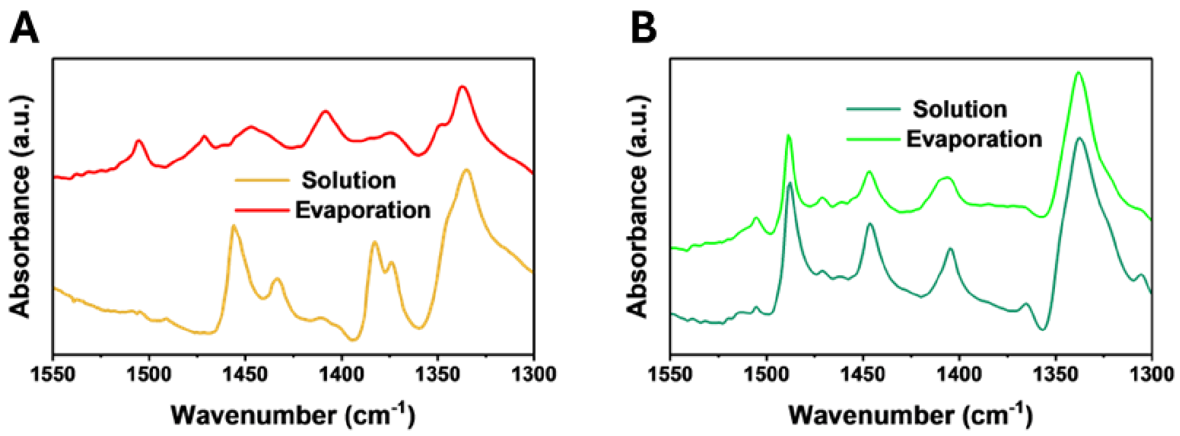

Supplementary Figure 37: Overlay of the solution processed and thermally evaporated thin films of **(A)** NDI-(EtPA)$_2$ and **(B)** NDI-(PhBr)$_2$.

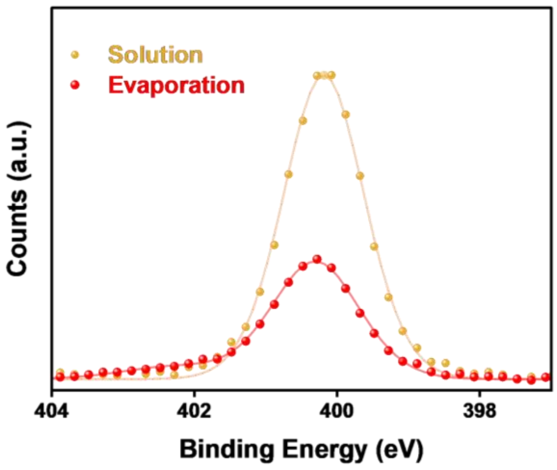

Supplementary Figure 38: N 1s XPS spectra for NDI-$(EtPA)_2$ processed via solution **(orange)** and evaporation **(red)**.

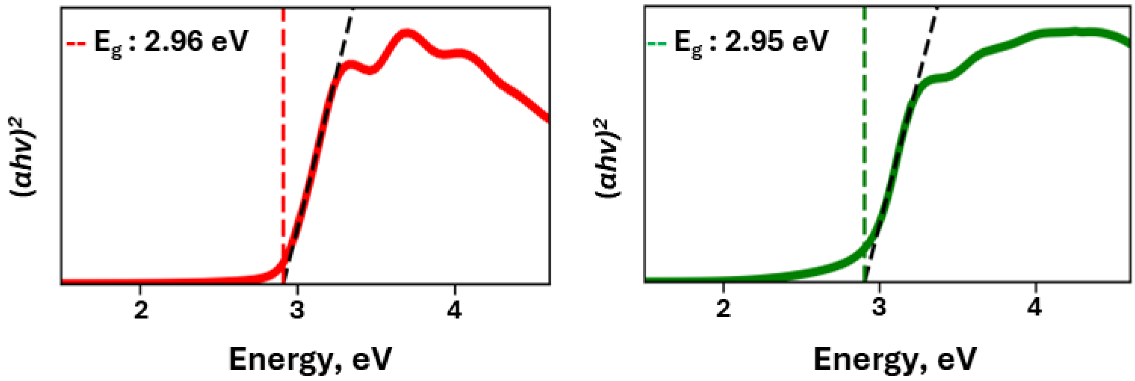

Supplementary Figure 39: Tauc plots of **(A)** NDI-$(EtPA)_2$ and **(B)** NDI-$(PhBr)_2$.

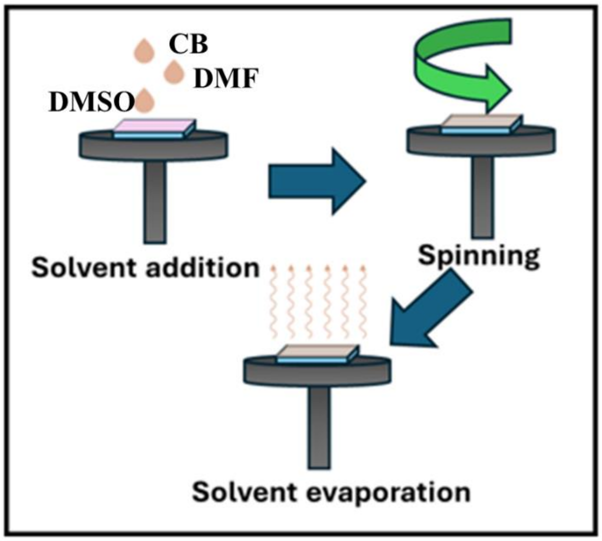

Supplementary Figure 40: Schematic of the solvent wash process undertaken to simulate the effects of solvents during perovskite deposition on top of our NDI films.

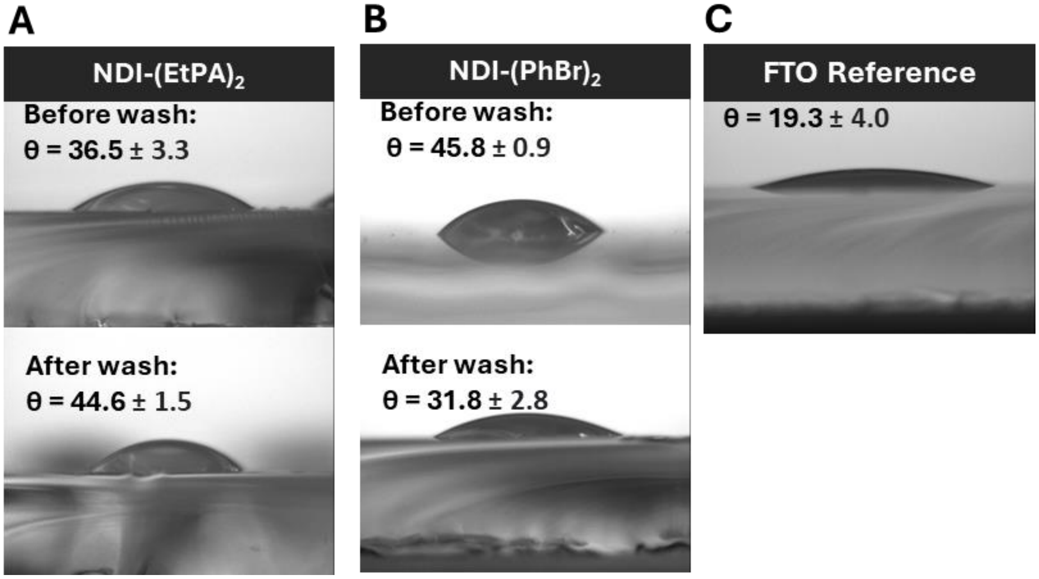

Supplementary Figure 41: Contact angle of **(A)** NDI-$(EtPA)_2$, **(B)** NDI-$(PhBr)_2$, and **(C)** FTO with H_2O.

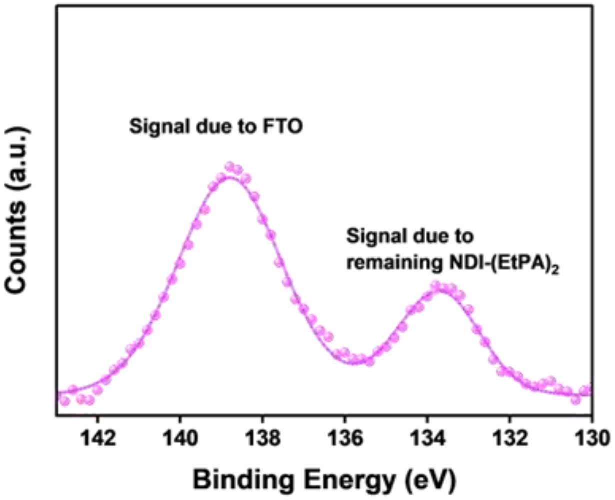

Supplementary Figure 42: P 2p XPS spectrum of an NDI-$(EtPA)_2$ film after the solvent wash process.

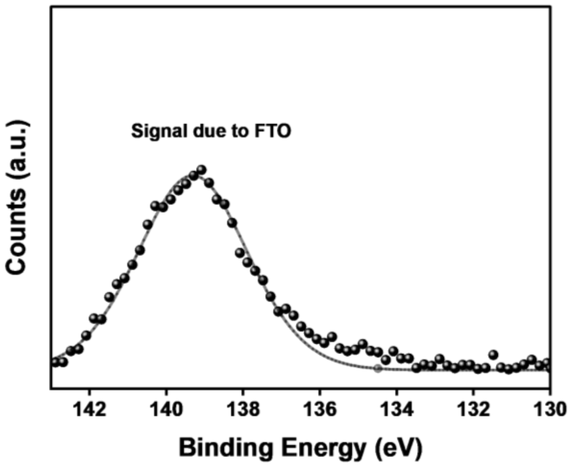

Supplementary Figure 43: P 2p XPS spectrum of bare FTO.

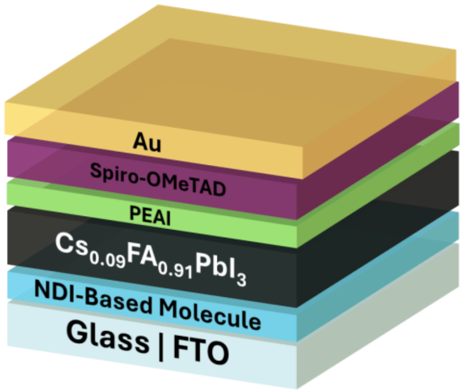

Supplementary Figure 44: Schematic of the perovskite solar cell architecture used in this work.

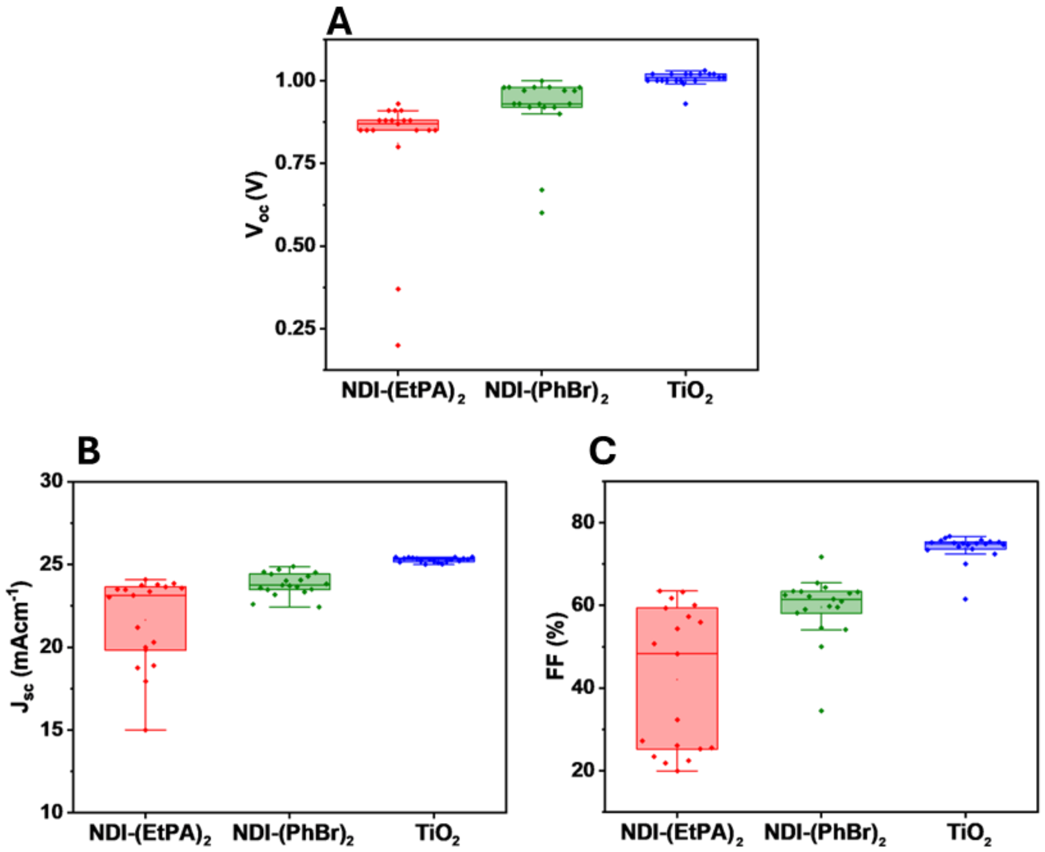

Supplementary Figure 45: Box plots depicting the distributions in **(A)** Voc, **(B)** Jsc, and **(C)** Fill Factor for NDI-(EtPA)$_2$, NDI-(PhBr)$_2$, and our TiO_2 reference. The solid points represent individual data points, while the hollow points represent the mean of a given distribution. The horizontal bar represents the median value of a distribution while the box signifies the interquartile range.

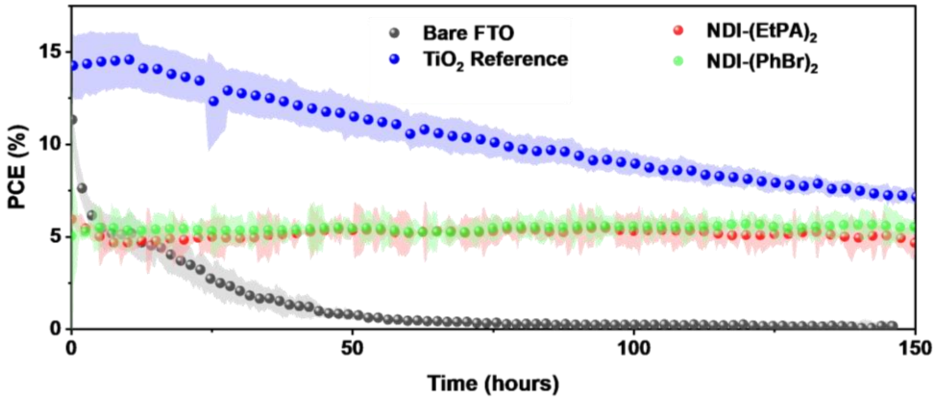

Supplementary Figure 46: Stabilized PCE of NDI-(EtPA)$_2$, NDI-(PhBr)$_2$, our TiO_2 reference devices, and devices without an ETL.

References:

1) Graillot, A.; Monge, S.; Faur, C.; Bouyer, D.; Robin, J.J. Synthesis by RAFT of Innovative Well-Defined (co) Polymers from a Novel Phosphorus-Based Acrylamide Monomer. *Polym. Chem*., **2013**, 4, 795. DOI: 10.1039/c2py20720f
2) Grabicki, N.; Dumele, O.; Sai, H.; Powers-Riggs, N.E.; Phelan, B.T.; Sangji, M.H.; Chapman, C.T.; Passarelli, J.V.; Dannenhoffer, A.J.; Wasielewski, M.R.; Stupp, S.I. Polymorphism and Optoelectronic Properties in Crystalline Supramolecular Polymers. *Chem. Mater*., **2021**, 33, 706-718. DOI:10.1021/acs.chemmater.0c04123.
